# Type-I interferons promote innate immune tolerance in macrophages exposed to *Mycobacterium ulcerans* vesicles

**DOI:** 10.1371/journal.ppat.1011479

**Published:** 2023-07-10

**Authors:** Quentin Bernard, Maïssa Goumeidane, Emmanuel Chaumond, Marie Robbe-Saule, Yan Boucaud, Lucille Esnault, Anne Croué, Jerome Jullien, Laurent Marsollier, Estelle Marion

**Affiliations:** 1 Univ Angers, Nantes Université, INSERM, Immunology and New Concepts in ImmunoTherapy, INCIT, UMR 1302, Angers, France; 2 Nantes Université, INSERM, CRTI, Nantes, France; 3 Laboratoire d’anatomo-pathologie, CHU Angers, Angers, France; University of Washington, UNITED STATES

## Abstract

Buruli ulcer is a chronic infectious disease caused by *Mycobacterium ulcerans*. The pathogen persistence in host skin is associated with the development of ulcerative and necrotic lesions leading to permanent disabilities in most patients. However, few of diagnosed cases are thought to resolve through an unknown self-healing process. Using *in vitro* and *in vivo* mouse models and *M*. *ulcerans* purified vesicles and mycolactone, we showed that the development of an innate immune tolerance was only specific to macrophages from mice able to heal spontaneously. This tolerance mechanism depends on a type I interferon response and can be induced by interferon beta. A type I interferon signature was further detected during *in vivo* infection in mice as well as in skin samples from patients under antibiotics regiment. Our results indicate that type I interferon-related genes expressed in macrophages may promote tolerance and healing during infection with skin damaging pathogen.

## Author summary

*Mycobacterium ulcerans*, the causative agent of Buruli ulcer, induces persisting ulcerative and necrotic lesions in the skin of patients. While most of them require a medical intervention to recover from the infection, few are thought to heal without treatment through an unknown self-healing process. We report here that the tissue healing is associated with a type-I interferon response *in vivo*. *In vitro*, a similar response that can be induced by interferon beta promotes tolerance in mouse macrophages. Our results indicate that type I interferon-related genes expressed in macrophages may promote tissue healing through an innate immune tolerance mechanism.

## Introduction

Buruli ulcer, a chronic infectious disease caused by *Mycobacterium ulcerans* (*M*. *ulcerans*), is the third most common mycobacterial disease worldwide after tuberculosis and leprosy. It is one of the 20 neglected tropical diseases listed by the World Health Organization (WHO). It affects hundreds of people every year, in 33 different countries, with foci in West and Central Africa and in Australia [1]. The route of *M*. *ulcerans* transmission remains unclear, but a few weeks after its inoculation into the human dermis it can cause a necrotizing hypodermitis, with the appearance of an early closed lesion, such as a nodule, plaque and/or edema, progressing to a late ulcerative lesion [2]. Most lesions are located on the upper or lower limbs and their extent varies considerably, ranging from a diameter of less than 5 cm to an area covering an entire limb.

The first major advance in our understanding of the pathogenesis of Buruli ulcer came with the discovery of a lipid toxin, mycolactone, secreted by *M*. *ulcerans* during infection [3]. Without this toxin, the mycobacterium cannot colonize its hosts or cause dermal injuries [4,5]. Mycolactone toxicity is dependent on the ability of this toxin to bind Sec61, a protein located in the endoplasmic reticulum (ER) membrane of host cells [6]. Sec61 binding enables mycolactone to block the protein translocation machinery involved in the transport of newly synthesized membrane and secreted proteins, inducing cell stress that eventually leads to activation of the transcription factor ATF4 and the activation of apoptosis [7]. The Sec61-mycolactone interaction also impedes the inflammatory response, as the toxin prevents cells from producing most secreted cytokines and membrane immune receptors [8]. This anti-inflammatory activity has been linked to the ability of the mycobacterium to persist within host tissues without clearance [9,10]. However, it is not consistent with the observed presence of a significant inflammatory cell infiltrate around the infection site observed in patients and infected-mouse lesions. We recently reconciled these observations by showing that mycolactone, either within *M*. *ulcerans* or in natural extracellular vesicles (MEVs) secreted during infection, also has inherent pro-inflammatory activity mediated by activation of the inflammasome and the Sec61-independent secretion of IL-1 family cytokines [11,12]. MEVs, which are known to be associated with mycobacterial pathogenesis [13], are detected in biopsy specimens from patients with active Buruli ulcer, suggesting a key role for these vesicles in spreading inflammation [14].

*M*. *ulcerans* infection is most commonly associated with the development of necrotic lesions in patients, leading in 25% of cases to permanent disabilities; however, about 5% of newly diagnosed cases are thought to resolve through a self-healing process [15,16]. In either case, the mycobacterium can remain in host tissues, detectable for several months, or even years, if left untreated. Such persistence of the pathogen in host skin suggests that immune cells are subject to repeated contact with mycobacterial antigens. However, the effect of these repeated interactions on the pathogenesis of Buruli ulcer is unknown. Repeated exposure to pathogen/damage associated molecular patterns (PAMPs/DAMPs) has been shown to induce a form of innate immune memory in the context of other bacterial diseases [17,18]. This memory is characterized by long-lasting antigen-non-specific epigenetic reprogramming events in innate immune cells, increasing (trained immunity) or decreasing (tolerance) the strength of the inflammatory response relative to the initial activation. Trained immunity, associated with an upregulation of inflammatory cytokines following repeated exposure to pathogen antigens, has been shown to protect against several infectious diseases, including those caused by *Streptococcus pneumoniae*, *Plasmodium*, *Leishmania*, *Toxoplasma gondii* and *M*. *tuberculosis* [19–23]. It has recently been suggested that a dysregulation of trained immunity may also contribute to the detrimental inflammation observed in bacterial infections such as Lyme disease or during atherosclerosis [24,25]. The opposing mechanism, innate immune tolerance, protects the host from detrimental outcomes induced by excessive inflammation. It was originally characterized in response to LPS, where prior challenge with a low dose of the toxin mediates host protection against sepsis [26,27]. Innate immune tolerance has since been studied in the context of host-microbiome interactions, in particular in atherosclerosis, rheumatoid arthritis, multiple sclerosis or diabetes [28–30]. Fine-tuning of the regulation of trained immunity and innate immune tolerance enables the host to develop a protective immune response while simultaneously avoiding overly exuberant responses that lead to host damage.

Our understanding of *M*. *ulcerans* infection has been bolstered by studies in mouse models, with BALB/c or C57BL/6 mouse strains effectively mimicking the pathogenesis of Buruli ulcer in humans. Subcutaneous inoculation of the tail or footpad of these mice with *M*. *ulcerans* leads to edema formation after several weeks, followed by ulceration and progression toward tissue necrosis and death. Infection stages are characterized by an accumulation of myeloid cells around the necrotic area and the production of large amounts of inflammatory cytokines, such as IL1β [31,32]. By contrast, another inbred mouse strain, FVB/N, exhibits spontaneous healing after the ulcerative stage without clearance of the mycobacterium, mimicking the self-healing process observed in a minority of patients [33]. This healing process is associated with an accumulation of macrophages in draining lymph nodes and a decrease in inflammatory cytokine expression. We hypothesize that repeated exposure to *M*. *ulcerans* antigens may favor tolerance over trained immunity specifically in macrophages from FVB/N mice, thereby preventing the pro-inflammatory response characteristic of BALB/c mice and instead leading to establishment of the healing process.

## Results

### FVB/N mice uniquely exhibit signs of tolerance upon repeated exposure to *M*. *ulcerans* vesicles

**Fig 1:**
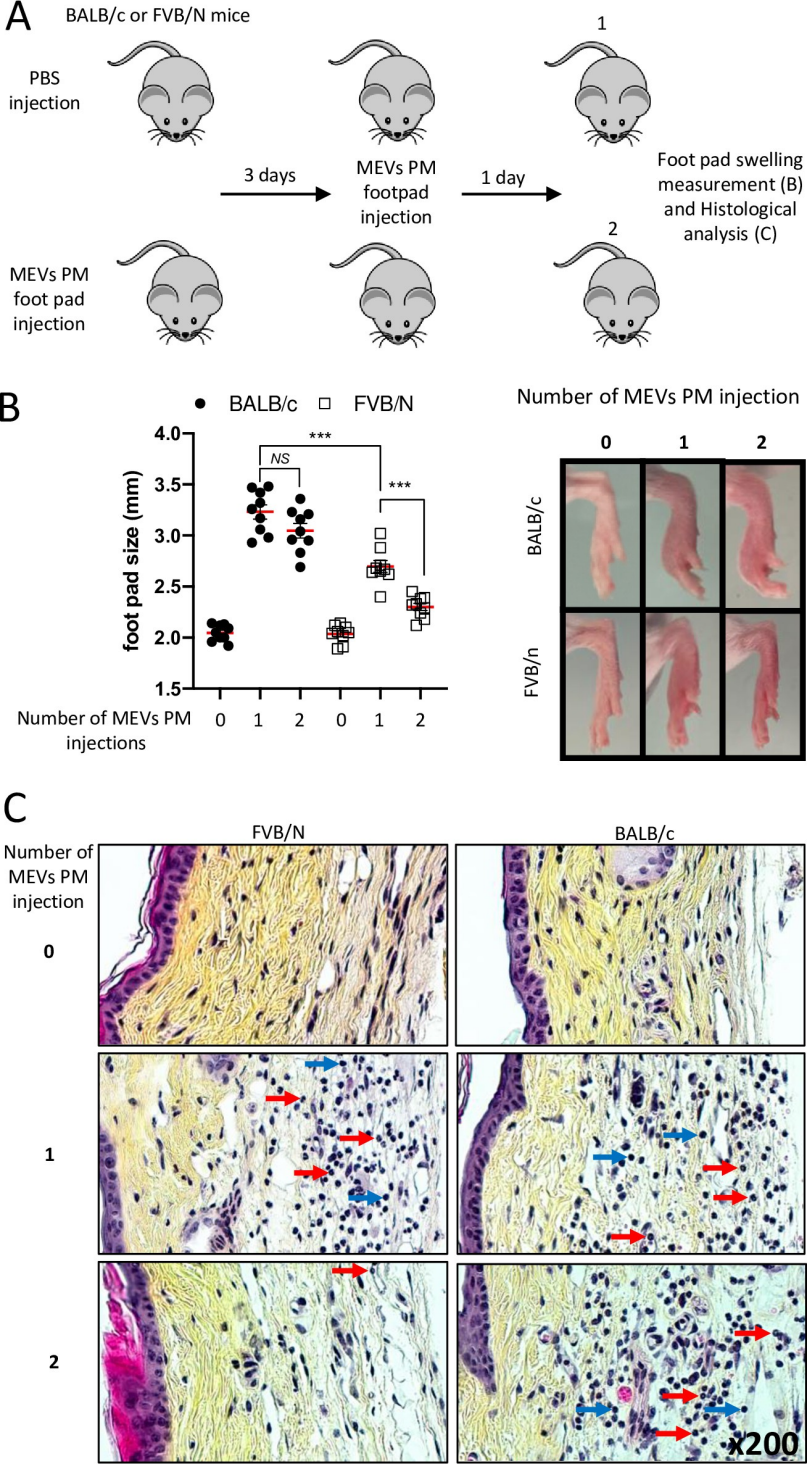
Repeated exposure to *M*. *ulcerans* vesicles containing mycolactone induces a tolerance phenotype in FVB/N but not in BALB/c mice. (**A**) A schematic representation of our footpad injection model. In the first group, BALB/c and FVB/N mice were injected with PBS, then three days later with *M*. *ulcerans* extra-cellular vesicles containing 2 ug of mycolactone (MEVs PM). In the second group, BALB/c and FVB/N mice were injected twice with MEVs PM three days apart. 24 hours following the last injection, the host immunological response was monitored. (**B**) Footpad swelling was determined using a caliper. (**C**) Tissues were collected for histological analysis by Hematoxylin-phloxine-saffron staining. Red and blue arrows are pointing towards polynuclear cells and mononuclear cells respectively. (**A**, **B** and **C**) 0, 1 and 2 refer to the mice that received PBS only, PBS then MEVs PM and MEVs PM twice respectively. Results are expressed as means ± SEM of three independent experiments (n = 3 for each condition in each independent experiment). Statistical analysis was performed using one-way ANOVA with Tukey post-test *(NS*: not significant, ***P < 0.001).

It is well established that about 35 days after subcutaneous infection (dpi) with *M*. *ulcerans*, both FVB/N and BALB/c mice develop edema with signs of ulceration. After 45 days, this ulceration progresses to irreversible necrosis in BALB/c mice, whereas it begins to regress and ultimately heals 90 dpi in FVB/N mice [33]. The duration of disease development implies that host cells are likely repeatedly exposed to *M*. *ulcerans* over the course of infection. We previously showed that the injection of MEVs purified from a *M*. *ulcerans* strain producing mycolactone (MEVs-PM) into mouse footpads induces an inflammatory response characterized by significant swelling in BALB/c mice. Inoculation with the same number of MEVs purified from a *M*. *ulcerans* strain unable to produce mycolactone (MEVs-NPM) does not result in skin damage [11]. We aimed to investigate whether sustained exposure to mycobacterium antigens affects the outcome of pathogenesis by analyzing the impact of multiple injections of MEVs-PM on the host inflammatory response. BALB/c and FVB/N mice received PBS or MEVs-PM footpad injections. Upon resolution of swelling, 3 days post-injection, all mice received a second injection with MEVs-PM (**Fig 1A**). FVB/N mice that received a single injection of MEVs-PM displayed less swelling than the equivalently treated BALB/c mice. Exposure to a second MEVs-PM injection induced a significantly weaker inflammatory response with less swelling compared to the initial exposure in FVB/N mice, but not in BALB/c mice, whose secondary response was not significantly different than their first (**Fig 1B**). We then performed a histological investigation of the treated footpads, to cellularly characterize the inflammation (**Fig 1C**). In either BALB/c or FVB/N mice, a single injection of MEVs-PM induces a significant recruitment of polynuclear cells to the dermis, as well as an increased number of mononuclear cells (macrophages/ lymphoid cells) as compared to PBS-treated mice (**Fig 1C**). In BALB/c mice, the second injection of MEVs-PM induces a pro-inflammatory immune response similar in magnitude and appearance to the first injection. In contrast, FVB/n mice injected a second time with MEVs-PM show a phenotype more closely resembling the control, with very few polynuclear cells recruited to the dermis. These observations suggest that resident cells from FVB/N mice develop a tolerance phenotype following repeated exposure to *M*. *ulcerans* vesicles containing mycolactone.

### Macrophages from FVB/N, but not BALB/c mice develop a tolerance phenotype following exposure to *M*. *ulcerans* vesicles plus mycolactone

**Fig 2:**
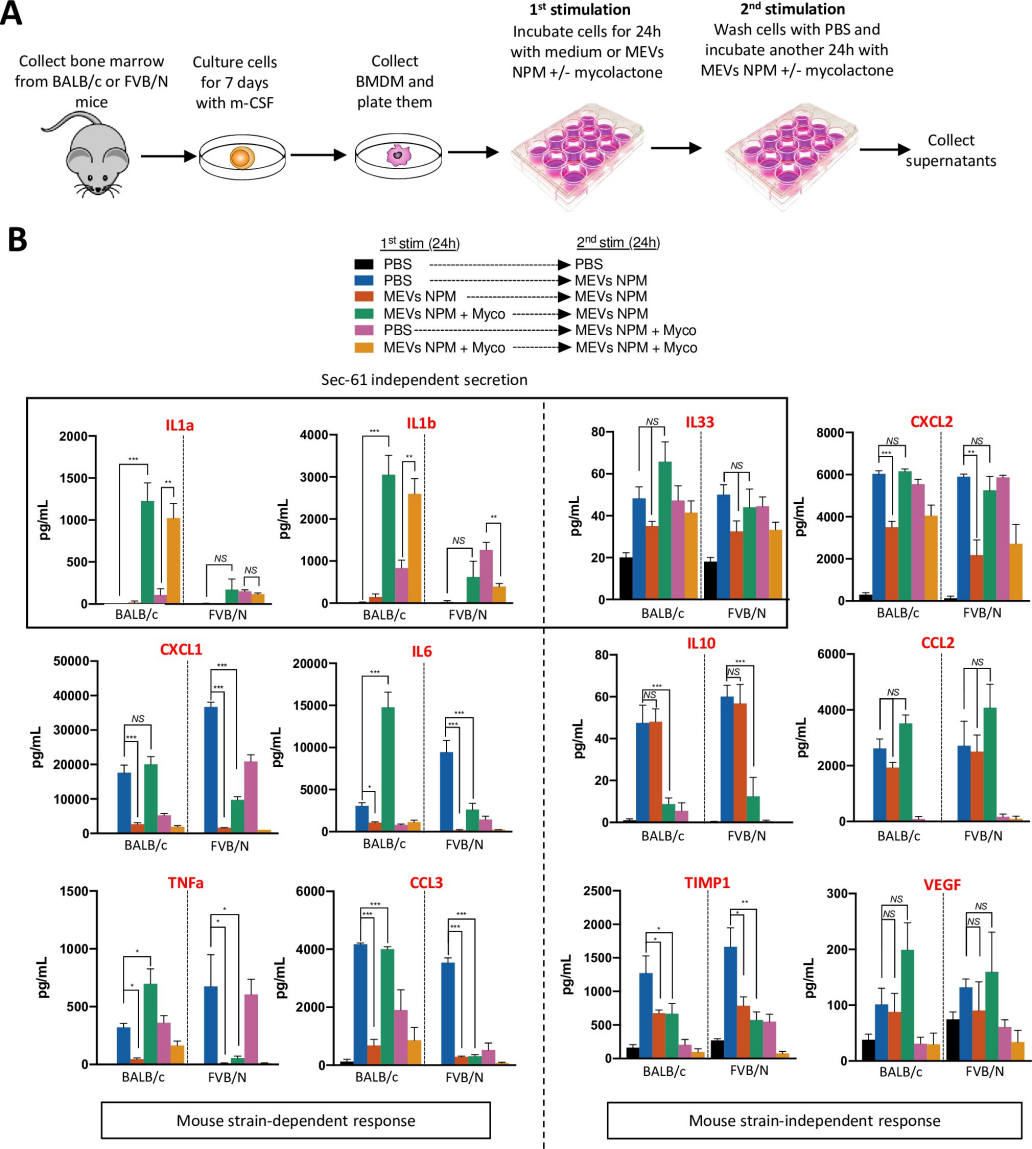
BALB/c but not FVB/N macrophages fail to develop tolerance against *M*. *ulcerans* vesicles in the presence of mycolactone. (**A**) A schematic representation of our *in vitro* protocol. The bone marrow from BALB/c and FVB/N mice was extracted. Collected cells were differentiated into macrophages using m-CSF for 7 days. Macrophages were seeded into plates and incubated for 24 hours with control media or media containing vesicles from a mycolactone deficient strain (MEVs NPM) (MOI: 20 000) +/- purified mycolactone (Myco) (6 ng/mL). Cells were washed with PBS and activated with the same ligands for another 24 hours at which point supernatants were collected for analysis. (**B**) Luminex analysis was performed to measure the levels of 12 inflammatory cytokines in the supernatant including IL1a and IL1b whose secretion is not dependent on Sec61 activity, and CXCL1, IL6, TNFa, CCL3, CCL2, CXCL2, IL10, IL33, TIMP1 and VEGF. Bars represent the mean ± SEM of at least three independent experiments. Statistical analysis was performed using one-way ANOVA with Tukey post-test (*NS* = not significant, *P < 0.05, **P < 0.005, ***p<0.001).

We focused on macrophage responses, as these cells are crucial immune sentinels of the skin and are present in the cell infiltrate in mouse tissues following *M*. *ulcerans* infection. Bone marrow was extracted from BALB/c and FVB/N mice and incubated with m-CSF for 7 days to promote macrophage differentiation. To differentiate between the effects of extracellular vesicles and of mycolactone, we incubated bone marrow-derived macrophages (BMDMs) for 24 hours in the presence or absence of MEVs-NPM, with or without the addition of purified mycolactone. We used a dose of 6 ng/mL mycolactone, as higher doses known to fully inhibit the secretion of IL-6 or TNFα resulted in a significant increase in cell death ([8,11,34] and S1 Fig). In agreement with previous studies (6,8,11), mycolactone decreased the production of several soluble mediators, including both inflammatory and regulatory cytokines and simultaneously increased the production of IL-1β by activated macrophages (Fig 2B).The cells were washed with PBS and restimulated with the same conditions for another 24 hours (**Fig 2A**). We then analyzed the cellular immune response with Luminex assays quantifying 12 different cytokines in the supernatant.

Following a single stimulation, the secretion profiles of BALB/c and FVB/N cells were similar. As expected, we observed significant levels of IL1a and IL1b, cytokines for which the secretion does not depends on Sec61 activity, induced in the presence of mycolactone, due to the ability of this toxin to activate the inflammasome (**Fig 2B, top left**) [11]. Moreover, lower levels of secretion for most MEV-NPM-induced soluble factors (CXCL1, IL6, CCL3, CCL2, IL10 and TIMP1) were induced in the presence mycolactone, due to inhibition of Sec61 by the toxin (**Fig 2B, blue vs. pink**).

The response of cells to two stimulations depends on the mouse strain of origin and the presence of mycolactone. Prior exposure to MEVs-NPM decreases the overall inflammatory response to a secondary MEV-NPM treatment independent of mouse strain (**Fig 2B, blue vs. orange**). This downregulation has been associated to innate immune tolerance in studies investigating the impact of successive TLR stimulations on the cellular inflammatory response [35,36]. Conversely, MEVs-NPM priming in the presence of mycolactone led to significant differences in the response to a secondary stimulation depending on the mouse strain as measured by secretion of six of the 12 cytokines tested (CXCL1, CCL3, IL-6, TNFα, IL-1a and IL-1b) (**Fig 2B**). When mycolactone was added during the first stimulation, FVB/N cells still showed reduced secretion of CXCL1, IL6, TNFα and CCL3 (**Fig 2B, blue vs. green**) and IL-1β (**Fig 2B, pink vs. yellow**) in response to subsequent MEVs stimulation, in contrast to increasing or unchanged levels in BALB/c cells. Under these conditions, secretion of IL1α did not decrease significantly in FVB/N cells, but the effects still contrasted with the increased secretion seen in BALB/c cells (**Fig 2B, pink vs. yellow**). These data suggest that, in the presence of mycolactone, BALB/c but not FVB/N BMDMs fail to maintain the tolerance phenotype normally observed following repeated exposure to *M*. *ulcerans* vesicles. In fact, BALB/c cells exacerbate the immune response for some cytokines, perhaps due to trained immunity. This difference seems to be specific to BMDMs, as similarly treated fibroblasts from BALB/c and FVB/N mice did not show strain-specific differences in IL-6 secretion, with both showing signs of tolerance (**S2 Fig**).

### Inflammatory tolerance in FVB/N macrophages in the presence of mycolactone corresponds to transcriptional differences

**Fig 3:**
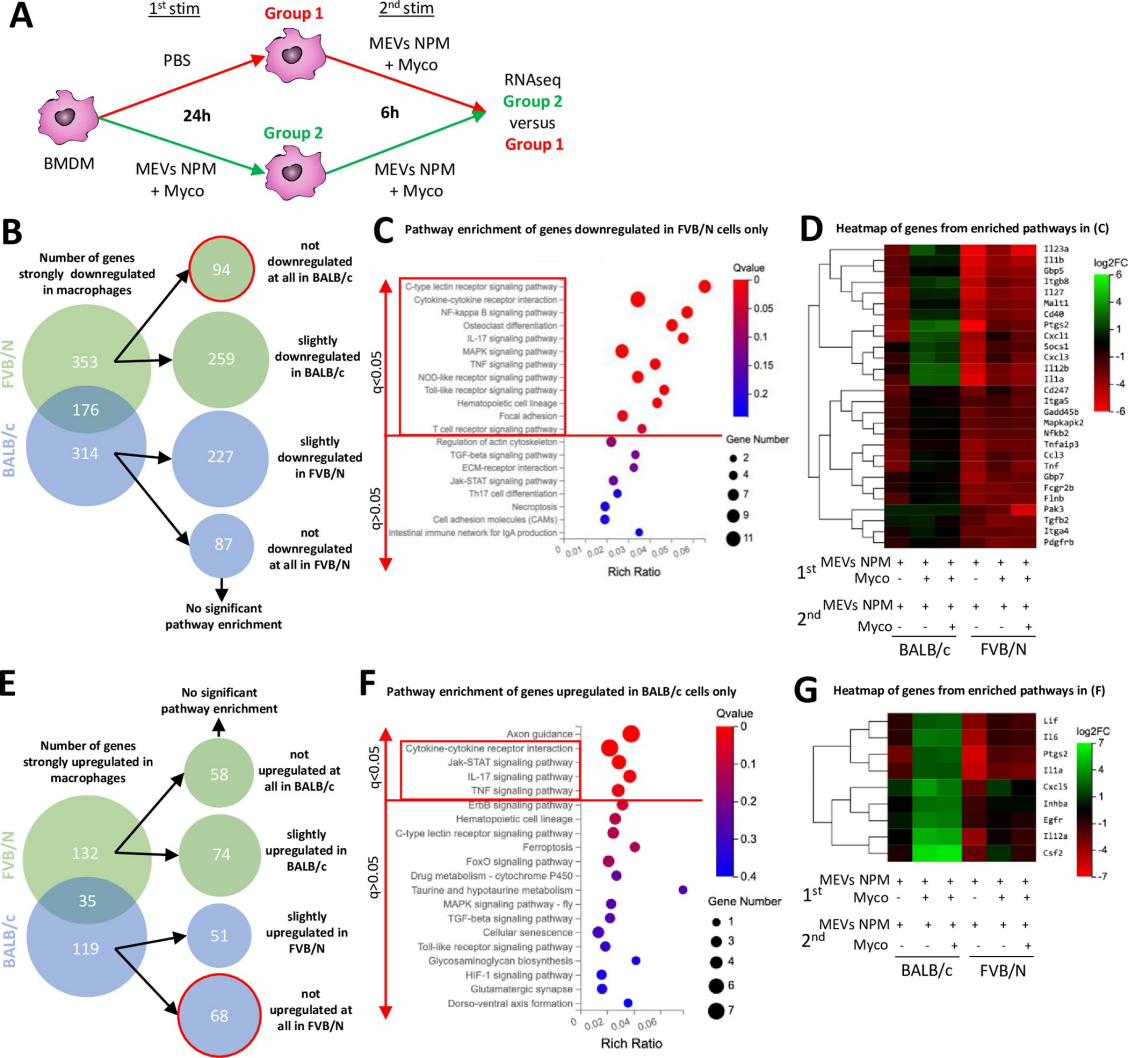
Repeated exposure to M. ulcerans vesicles with mycolactone differentially rewires the expression of selected immune genes in BALB/c or FVB/N macrophages. **(A)** A schema of our experiment setup: Macrophages from FVB/N and BALB/c mice were seeded into plates and incubated for 24 hours +/- vesicles from a mycolactone deficient strain (MEVs NPM, MOI: 20000) with purified mycolactone (Myco, 6 ng/mL). Cells were washed with PBS and activated a second time with the same ligands for six hours. mRNA was collected and sequenced. **(B)** Differential gene expression summary comparing cells stimulated twice with MEVs NPM + Myco vs. cells stimulated only once, for each mouse strain. Strongly downregulated genes have log2FC < = -2, Q value < = 0.05. Downregulated genes for each strain are subdivided into those downregulated slightly (-2 < log2FC < -0.5) and those not downregulated (log2FC > = -0.5) in the other strain. **(C)** Pathway enrichment of downregulated genes in FVB/N macrophages only. **(D)** Heatmap of genes included in the downregulated pathways from FVB/N macrophages (red square in **(C)**). **(D)** Upregulated gene expression summary comparing cells as in **(B)**. Categorization uses same log2FC and Q values as in **(B)** with positive log2FC values. **(F)** Pathway enrichment of upregulated genes in BALB/c macrophages only. **(G)** Heatmap of genes included in the upregulated pathways from BALB/c macrophages (red square in **(F)**). The Rich ratio is the ratio of the number of differentially expressed genes annotated in this pathway relative to all genes annotated in this pathway. A Q value is the corrected p value ranging from 0 to 1. Q values < 0.05 are considered significant. Data are based on three independent experiments.

We investigated whether immune response differences after repeated antigen exposure could be characterized at the transcriptional level. We used RNA sequencing of FVB/N and BALB/c cells stimulated twice with MEVs-NPM plus mycolactone compared to cells stimulated once with the same ligands (**Fig 3A**). We focused on the data obtained in the presence of mycolactone, corresponding to natural infection conditions. We observed a strong and significant decrease in expression (logFC<2, *q*<0.05) for 529 genes in FVB/N macrophages and 490 genes in BALB/c cells (**Fig 3B, S1 Data**). These genes included 94 that were exclusively downregulated in FVB/N macrophages and 87 that were only downregulated in BALB/c cells (**Fig 3A**). Interestingly, significant pathway enrichment was present only for the downregulated genes in FVB/N cells (**Figs 3C, S3A-S3B**), including multiple pro-inflammatory pathways, such as those regulating cytokine expression that are consistent with our Luminex secretion data (*e*.*g*. IL1α, IL1β, CXCL1, TNFα and CCL3) (**Figs 3D, S4**).

We also investigated the genes for which expression increased in cells exposed twice to MEVs-NPM plus mycolactone compared to cells exposed once. A strong, significant upregulation (logFC>2, *q*<0.05) was observed for 167 genes in FVB/N macrophages and 154 genes in BALB/c cells. These included 58 genes that were exclusively and strongly elevated in FVB/N macrophages and 68 that were only highly upregulated in BALB/c cells (**Fig 3E, S1 Data**). Significant pathway enrichment for the upregulated genes was observed only in BALB/c macrophages (**Figs 3F, S3C-S3D**), with pathways involving pro-inflammatory cytokines, consistent with the Luminex analysis, such as IL1α and IL6 (**Figs 3G, S4**). Thus, the transcriptomic profile of mouse macrophages after two stimulations corresponds to the cytokine secretion profiles of these cells, with inflammation mostly dampened in FVB/N cells and enhanced in BALB/c cells at the RNA and protein levels.

### The induction of interferon-stimulated genes (ISGs) following initial exposure to *M*. *ulcerans* vesicles and mycolactone allows to maintain immune tolerance

**Fig 4:**
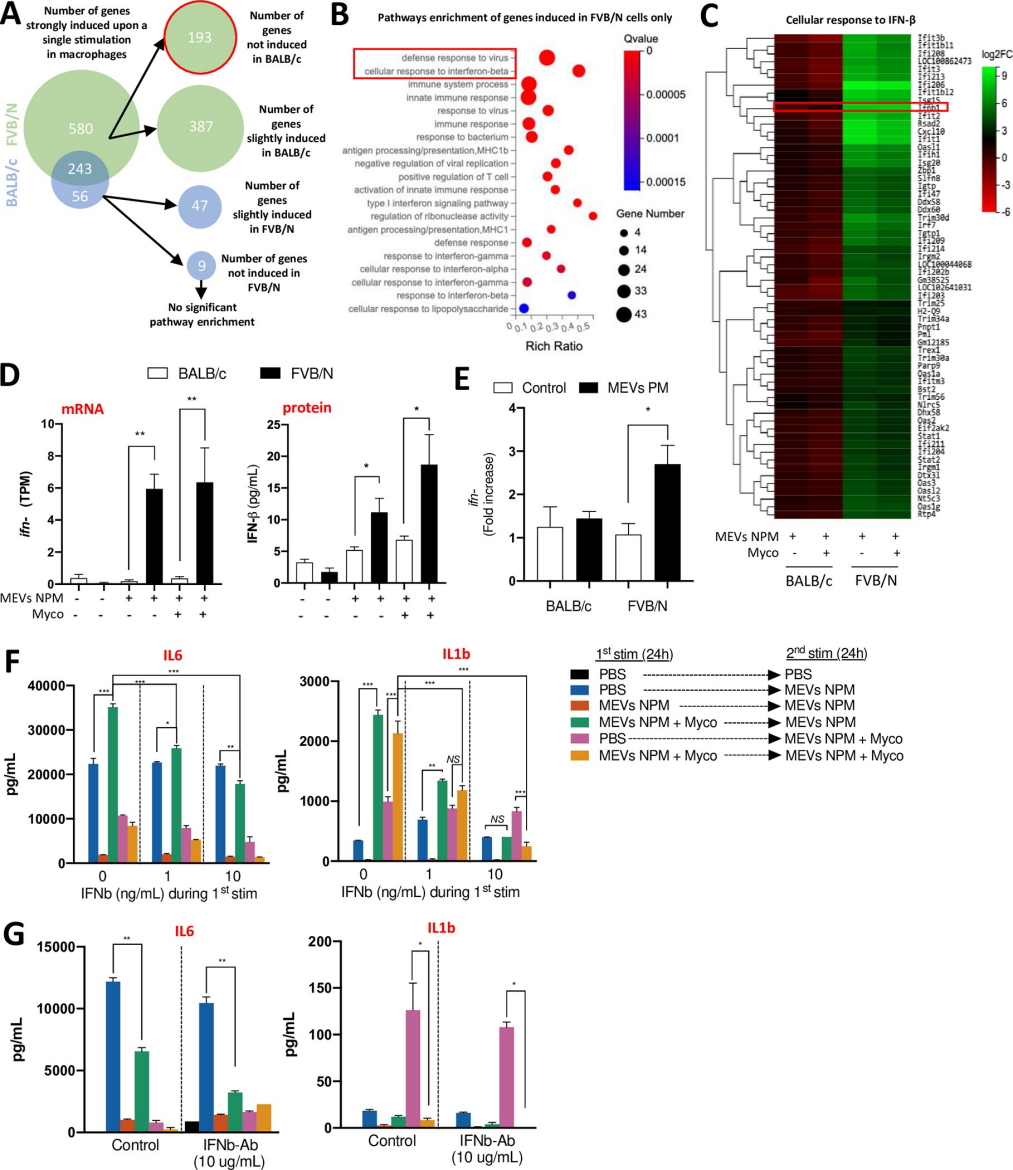
Tolerance developed in macrophages following initial exposure to *M*. *ulcerans* vesicles and mycolactone is allowed by the induction of type-I interferon related genes. Macrophages from FVB/N and BALB/c mice were seeded into plates and incubated for six hours +/- vesicles from a mycolactone deficient strain (MEVs NPM, MOI:20,000) in the presence of purified mycolactone (Myco, 6 ng/mL). mRNA was collected and sequenced. **(A)** Representation of upregulated differential gene enrichment following single incubation with vesicles and mycolactone as compared to untreated cells. Upregulated genes have log2FC > = 2, Q value < = 0.05. Slight induction is defined as (2 > log2FC > 0.5). Not induced is defined as log2FC < 0.5, Q value < = 0.05. **(B)** Pathway enrichment of genes induced in FVB/N macrophages only. **(C)** Heatmap of the genes induced in FVB/N macrophages belonging to the cellular response to IFN-β pathway. **(D)** The expression level of *ifnb* was measured at the mRNA level (Left panel) by RNAseq and at the protein level (Right panel) by ELISA. **(E)** The expression level of *ifnb* in the mouse footpad of BALB/c and FVB/N mice following a single injection of MEVs PM measured by qPCR. **(F)** BALB/c macrophages were seeded into plates and incubated for 24 hours +/- vesicles from a mycolactone deficient strain (MEVs NPM, MOI: 20,000) +/- purified mycolactone (Myco, 6 ng/mL), with or without recombinant IFN-β at the indicated concentrations. Cells were washed with PBS and activated by vesicles +/- mycolactone for another 24 hours before supernatants were collected and the secretion of IL1b and IL6 was measured by ELISA. **(G)** An analogous experiment using FVB/N macrophages and a IFN-β blocking antibody instead of IFN-β protein was performed. The legend is the same as in **(F)**. (**D-E**) Bars represent the mean ± SEM of three independent experiments. (**F-G**) Bars represent the mean ± SEM of two independent experiments. **(A-C)** The Rich ratio is the ratio of the number of differentially expressed genes annotated in this pathway relative to all genes annotated in this pathway. A Q value is the corrected p value ranging from 0 to 1. Q values < 0.05 are considered significant. **(D-G)** Statistical analysis was performed using one-way ANOVA with Tukey post-test (*NS* = not significant, *P < 0.05, **P < 0.005, ***P >0.001).

To investigate why macrophages from FVB/N mice could reduce the expression of selected inflammatory genes in the presence of mycolactone while macrophages from BALB/c mice could not, we performed RNAseq to characterize the cellular response to the first stimulation. No significant differences in gene expression were observed when comparing induction with MEVs-NPM alone and with mycolactone for either strain (**S5A Fig**). This suggests that the inability of BALB/c macrophages to reduce the production of selected inflammatory factors in the presence of mycolactone is dependent on post-transcriptional regulatory events. Nevertheless, a significantly larger number of genes were induced exclusively in FVB/N macrophages (193) than in BALB/c cells (9) upon initial stimulation (**Figs 4A, S5B, S1 Data**). We saw pathway enrichment only for genes induced in FVB/N cells, particularly including type I interferon-related genes such as *ifn-b*, *stat1/2*, *irf7*, *ifit1-3* and *isg15* (**Fig 4B**). IFN-β, the main upstream inductor of this pathway, was significantly upregulated in FVB/N macrophages both at the mRNA and protein levels (**Fig 4C, 4D**). Interestingly, despite the ability of mycolactone to inhibit Sec61 secretion activity, IFN-β was secreted in significantly higher concentrations by FVB/N than by BALB/c cells even in its presence (**Fig 4D**). Furthermore, similar observations were made in our *in vivo* model of vesicles injection as the expression of IFN-β was significantly increased in the footpad of FVB/N but not BALB/c mice injected with MEVs-PM as compared to PBS injected mice (**Fig 4E**). These data, together with a previous report showing a role of IFN-β and ISGs in downregulating the inflammatory response in specific conditions [37], suggest that the ability of FVB/N cells to activate the type I interferon pathway following the first stimulation may allow these cells to maintain a tolerance phenotype despite the presence of mycolactone, in contrast to BALB/c macrophages.

To investigate that hypothesis, we explored the ability of the type I interferon pathway to dampen the macrophage inflammatory response during repeated exposure to MEVs-NPM plus mycolactone, by supplementing BALB/c macrophages with various concentrations of IFN-β during the first stimulation. Cells were then washed and stimulated with fresh MEVs-NPM in the presence or absence of mycolactone, and the secretion of two key inflammatory cytokines was assessed by ELISA. The addition of IFN-β during the first stimulation restored the ability of BALB/c macrophages to tolerize the induction of IL-1b and IL-6 in a dose-dependent manner in the presence of mycolactone (**Fig 4F**, **blue vs. green and yellow vs. pink**). These findings confirm that inducing the cellular pathway of type-I interferons can act as negative regulator of the inflammation induced by *M*. *ulcerans* antigens. In parallel, to assess the requirement for IFN-β itself in the tolerance phenotype, we explored the ability of FVB/N macrophages to tolerize the secretion of inflammatory cytokines in the presence of IFNAR-blocking antibody during the first stimulation. Unexpectedly, we found that blocking the IFN-β receptor did not abolish the ability of FVB/N macrophages to tolerize the secretion of IL1β (**Fig 4G, blue vs. green and yellow vs. pink**). This finding suggested that either the first wave of IFN-β or ISGs, that can be induced directly through TLR2 activation [38], promote tolerance via an IFNAR-independent pathway in FVB/N macrophages.

### FVB/N macrophage immune tolerance phenotype *in vitro* is associated with histone methylases

**Fig 5:**
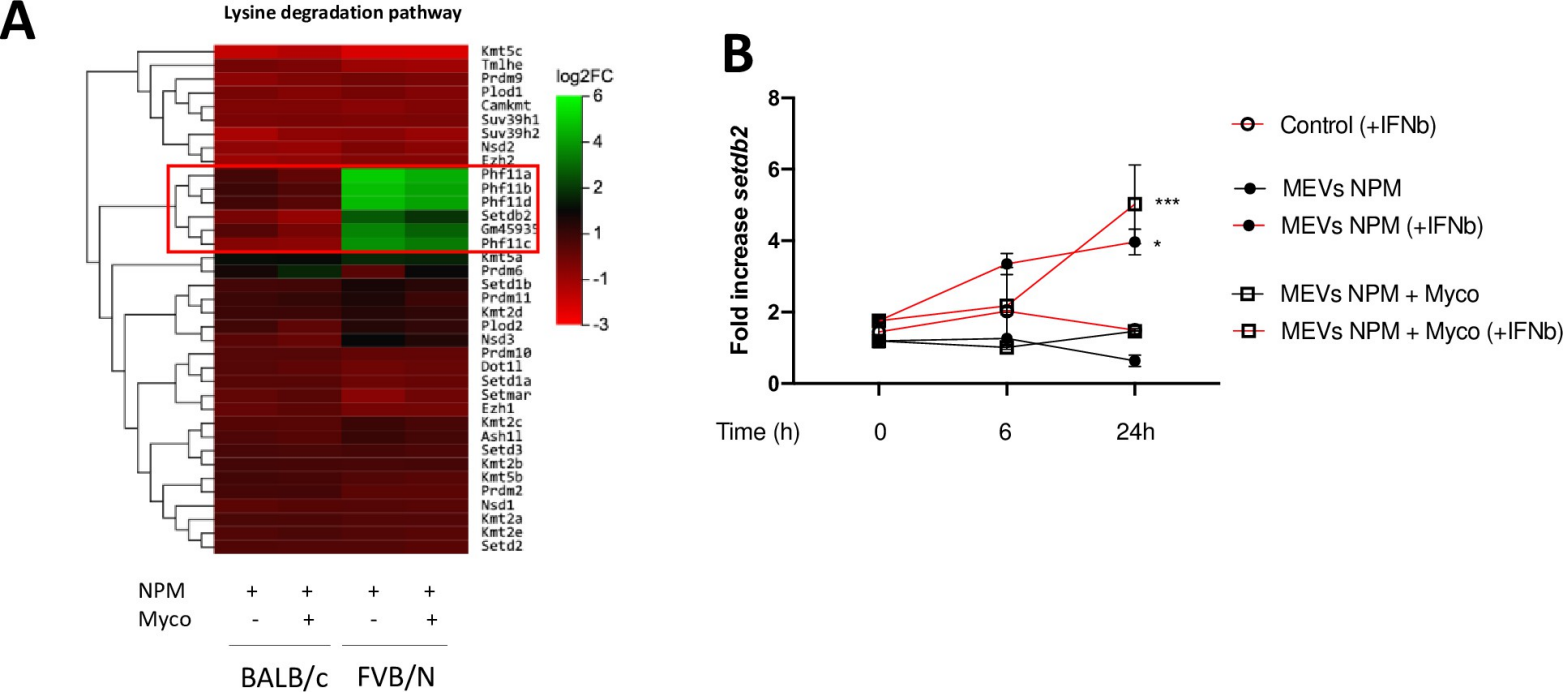
The induction of innate immune tolerance in the presence of mycolactone in FVB/n macrophages is associated with histone methylases. Macrophages from FVB/N and BALB/c mice were seeded into plates and incubated or not for six hours with vesicles from a mycolactone deficient strain (MEVs NPM) (MOI: 20 000) in the presence of purified mycolactone (Myco) (6 ng/mL). mRNAs were collected and gene expression was measured by RNAseq. (**A**) A heatmap of genes related to the lysine degradation pathway including most of the enzymes involved in the methylation of histone’s lysine residues. (**B**) Macrophages from BALB/c mice were seeded into plates and incubated or not for 6 or 24 hours with vesicles from mycolactone deficient strain (MEVs NPM) (MOI: 20 000) +/- purified mycolactone (Myco) (6 ng/mL) and recombinant IFN-β (1 uM). mRNAs were collected and gene expression was measured by qRT-PCR. The expression of *setdb2* is shown as fold change over the control condition (medium only). **(A)** Data are based on three independent experiments. **(B)** Data represent the mean ± SEM of two independent experiments. Statistical analysis was performed using one-way ANOVA with Tukey post-test (*P < 0.05, ***P >0.001).

The induction of innate immune memory relies on the presence of epigenetic marks deposited on histone proteins [17]. These modifications, most often methylation and acetylation of lysine residues, control chromatin access to the transcriptional machinery, thus regulating gene expression. Type I interferons have been shown to influence epigenetic regulation in macrophages, leading to tolerance through an upregulation of the *setdb2* lysine methyltransferase gene [39]. Our RNAseq analysis revealed that among the genes encoding histone modification enzymes, *setdb2* and few other genes from the same locus were upregulated in FVB/N but not BALB/c cells following the first stimulation with *M*. *ulcerans* antigens (**Fig 5A, S1 Data**). SETDB2 is an enzyme involved in the methylation of H3K9me2 to H3K9me3, a mark mostly present in inter-genic regions and associated with transcriptional repression. In BALB/c macrophages, the setdb2 induction was restored when recombinant IFN-β was added in the presence of MEVs NPM (**Fig 5B**). Together, these data suggest that methylases, and the SETDB2 pathway, are active even in the presence of mycolactone and could maintain the development of innate immune tolerance observed in FVB/N macrophages following repeated exposure to *M*. *ulcerans* antigens.

### A type-I interferon response is associated with healing conditions during *M*. *ulcerans* infection

**Fig 6:**
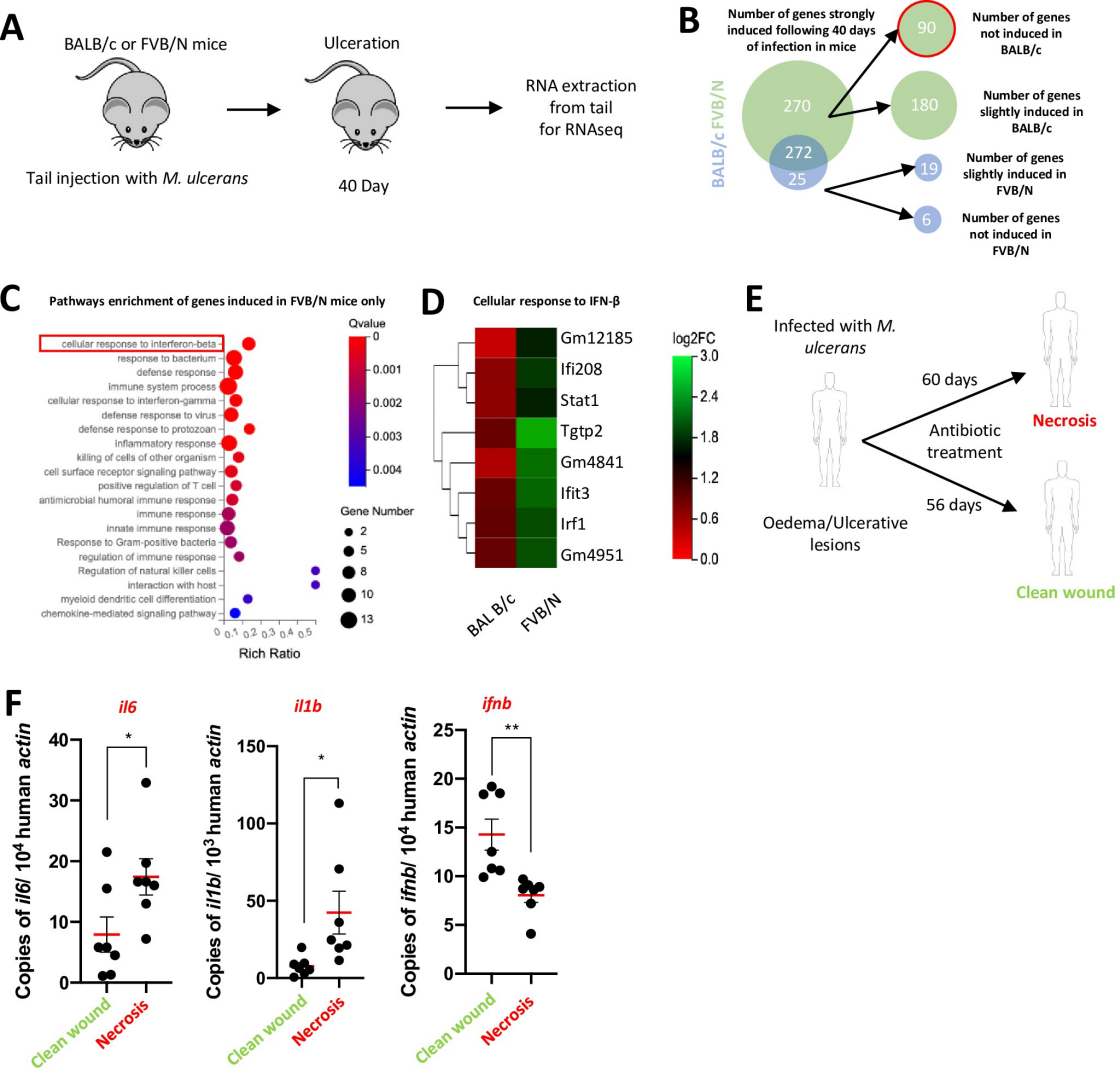
An interferon beta response is associated with healing conditions during *M*. *ulcerans* infection. **(A)** BALB/c and FVB/N mice were injected in the tail with living *M*. *ulcerans* organisms. 40 days following the infection, when the ulceration stage was reached, mice were sacrificed and the skin from the injection site was collected. mRNAs were purified and gene expression was measured by RNAseq. **(B)** Representation of upregulated differential gene enrichment in infected mice as compared to naïve mice. Upregulated genes have log2FC > = 1.5, Q value < = 0.05. Slight induction is defined as (1.5> log2FC > 0.5). Not induced is defined as log2FC < 0.5, Q value < = 0.05. **(B)** Pathway enrichment of genes induced in FVB/N mice only. **(C)** Heatmap of the genes induced in FVB/N macrophages belonging to the cellular response to IFN-β pathway. **(E)** Skin biopsies from patients infected by *M*. *ulcerans* were collected during the antibiotic treatment. Samples were separated into two groups based on the phenotype of their lesions. Patients showing advanced signs of healing were categorized as “clean wound” while patients still showing a highly necrotic lesion were categorized as “necrotic”. The tissue collection happened after a median duration of treatment of 60 and 56 days for the necrosis and clean wound group respectively. mRNAs were collected and gene expression was measured by qRT-PCR. (**B-D**) The Rich ratio is the ratio of the number of differentially expressed genes annotated in this pathway relative to all genes annotated in this pathway. A Q value is the corrected p value ranging from 0 to 1. Q values < 0.05 are considered significant. Data are based on 6 and 7 individual samples per group in (**A-D**) and (**E-F**) respectively. **(F)** Scatter plots represent the mean ± SEM Statistical analysis was performed using t-test (*P < 0.05, **P < 0.005).

Based on our *in vitro* experiments, we made the hypothesis that the induction of the type I interferons pathway following infection with *M*. *ulcerans* could pave the way for the anti-inflammatory phenotype observed in FVB/N mice during the healing phase. We then collected skin tissues from BALB/c and FVB/N mice infected with *M*. *ulcerans* at the ulcerative stage, several days before the healing phase starts in FVB/N mice and performed RNAseq (**Fig 6A**). A significantly larger number of genes were induced exclusively in FVB/N tissues (90) than in BALB/c skin (6) upon infection at the ulcerative stage (**Figs 6B, S6A, S1 Data**). We saw pathway enrichment only for genes induced in FVB/N mice (**Figs 6C, S6B**), particularly including type I interferon-related genes such as *stat1*, *irf1*, *ifit3 and ifi208* (**Fig 6D**).

We then decided to investigate whether similar observations could be made in human tissues. Since samples from untreated patients were not available for ethical reasons, we measured the expression of selected inflammatory genes in skin biopsies collected at different stages during the antibiotic treatment. Of note, during the treatment, patients transition at different speed from a severe necrosis associated with inflammation toward clean wounds characterized by signs of tissue healing. During or after antibiotic treatment, some patients required surgery (for debridement, curettage, preparation for grafting, skin grafting). In the report of the surgical operation, the surgeon indicated the state of the wound, i.e. the massive presence of necrosis or a clean wound, with or without the need of a skin graft. The information was collected to categorize the lesions in two groups: necrosis (n = 7) versus clean wound (n = 7). The patients presented a paradoxical reaction was excluded from this study. (**Fig 6E, S1 Table**). The median duration of antibiotic treatment at the tissue collection date was similar between the two groups with 60 days for the necrosis group and 56 days for clean wound group (**S1 Table**). qPCR analysis performed on these samples revealed that while pro-inflammatory genes such as *il1b* and *il6* were significantly upregulated in the necrosis group as compared to the clean wound group, the opposite observation was made for *ifnb*, the main regulator of the type I interferon pathway (**Fig 6F**). Altogether, these data strongly associate the activation of the type I interferon pathway with the healing of *M*. *ulcerans* induced lesions.

## Discussion

The key feature of Buruli ulcer is the late development of necrotic lesions in cutaneous tissues. Most patients seek medical assistance when the disease is at an active inflammatory stage, but about 5% of Buruli ulcer cases present signs of spontaneous healing without prior clinical intervention [15]. The mechanisms underlying this spontaneous healing are currently unknown. Our results, using mouse models with differing infection course, indicate that type I interferon-related genes expressed in macrophages may promote tolerance and healing.

*M*. *ulcerans* infection leads to tail ulceration and necrosis in BALB/c mice, but FVB/N mice display spontaneous healing. We show here that macrophages from BALB/c and FVB/N mice develop a tolerance phenotype upon stimulation with MEVs-NPM alone. However, when the mycolactone is added to the vesicles, FVB/N cells only maintain a tolerance phenotype for several tested cytokines whereas BALB/c cells fail to do so and in fact exhibit signs of trained immunity instead. IL-1β and other members of the IL-1 family can promote trained immunity in monocytes and NK cells [40]. Both FVB/N and BALB/c cells express IL-1β after initial exposure to *M*. *ulcerans* vesicles and mycolactone; however, the absence of type I IFN production in BALB/c cells to counter this IL-1β may explain the development of trained immunity over tolerance in this strain [41]. Of note, the maintenance of the FVB/N cell tolerance phenotype despite the presence of mycolactone is associated with the presence of a type I interferon response, with IFN-β and IFN-stimulated genes (ISGs) being induced during the initial priming events. Crucially, despite the presence of mycolactone, supplementation with recombinant IFN-β results in the development of tolerance in macrophages from BALB/c mice as well, suggesting a major role played by the type-I interferon response in maintaining the tolerance in the presence of the toxin. However, tolerance cannot be abolished by blocking the interferon receptor (IFNAR) in FVB/N macrophages. Previous studies of viral infections have revealed the existence of IFNAR-independent pathways mediating the expression of ISGs, such as *IFIT1*, *IFIT2*, *IFIT3* and *ISG15* [42], all of which were expressed by FVB/N macrophages during priming with *M*. *ulcerans* antigens. IFIT1 has been shown to promote ISG expression and to decrease inflammation (as measured by TNFα and IL1b) in macrophages stimulated with LPS, via a mechanism involving histone deacetylation [43]. Besides, the activation of TLR2, the major TLR involved in the recognition of M. ulcerans vesicles [11], has been previously shown to induce the expression of type-I interferons as well [38]. Also, another study has shown that the IFNAR feedback loop is not strictly necessary for robust type I IFN expression in the context of viral infection [44]. We therefore hypothesized that either the first wave of IFN-β or ISGs induced in an IFNAR pathway-independent manner may promote macrophage tolerance to *M*. *ulcerans* despite the presence of mycolactone.

Type I interferons were originally reported to have antiviral activity via induction of ISGs [45,46]. During bacterial infection, type I IFNs can induce pro- or anti-inflammatory signals. It depends on various parameters, including the pathogen, the tissue, and interferon secretion kinetics and concentration [47–49]. For example, It can promote inflammation thus conferring protection during Pneumococcal Invasive Disease [50]. On the other hand, type I IFNs are also able to downregulate the inflammation, notably through the induction of IL-1Ra–an antagonist of the IL-1β receptor–and IL-10 [51], or by inhibiting the activation of the NLRP3 inflammasome [52]. This anti-inflammatory property of type I IFNs can be detrimental for the host. It is the case in tuberculosis, where it promotes *M*. *tuberculosis* infection by negatively controlling the secretion of IL-1β [53]. However, it can also be beneficial as IFN-β has been shown to promote resolution of the inflammatory response in mice during *E*. *coli*-induced pneumonia [54]. We show here that prior exposure to IFN-β in the presence of *M*. *ulcerans* antigens promotes the development of a tolerant phenotype for several pro-inflammatory cytokines in macrophages *in vitro*. Moreover, we observe the induction of a type I interferon pathway in FVB/N mice infected with *M*. *ulcerans* several days before signs of healing. This pathway, by promoting an anti-inflammatory environment, could promote the resolution of the inflammation required for the healing process [55] observed in FVB/N mice.

It is generally accepted that phenotypic states of macrophages, such as those leading to tolerance or trained immunity, are controlled through chromatin regulation mediated by methylation or acetylation of histones [17]. Type I interferons can lead to tolerance through *setdb2*, a lysine methyltransferase gene [39]. Another study has shown that SETDB2 expression in wound macrophages is upregulated by IFN-β, whereas the IFN-β -SETDB2 axis is impaired in diabetes, leading to a persistent inflammatory macrophage phenotype [56]. We also observed an IFN-β dependent induction of *setdb2* expression in macrophages before the acquisition of the tolerant phenotype. In this context of infection, a kinetic analysis of the macrophage epigenomic status could highlight the regulation link between macrophage tolerance and the type-I interferon response [57].

We showed that only macrophages from mice able to control excessive inflammation during *M*. *ulcerans* infection were able to produce type I IFNs in response to this pathogen *in vitro*. In humans, a small proportion of the patients infected with *M*. *ulcerans* also recover from the infection-induced tissue damage without treatment. The host genetic component of this phenomenon is intriguing, but largely unknown. One study found a microdeletion on chromosome 8 encompassing a long non-coding RNA gene (close to a cluster of defensin genes), in a familial form of severe Buruli ulcer [58]. Moreover, a specific allele of the ATG16L1 gene (rs2241880), producing a nonfunctional protein that impairs autophagy, has been associated with protective effects against Buruli ulcer [59]. This variant has also been characterized as a marker of protection against *Citrobacter rodentium* infection, as the associated impairment of autophagy results in the induction of a protective type I IFN response by the gut microbiota [60]. We show here, that in patients under antibiotic treatment, the closer they are from healing, the higher and the lower are the expression of *ifnb* and *il1b*/*il6* respectively. These data characterize IFN-β as a marker of healing in patients infected with *M*. *ulcerans*. Based on our mouse data, we make the hypothesis, that in humans, type I interferons could help promote the healing of *M*. *ulcerans* induced lesions by negatively regulating the inflammatory response through innate immune tolerance.

The link between type I IFN and innate immune tolerance is an interesting open question in the context of Buruli ulcer. In addition to host genetic studies, it would be of interest to also collect patient health status information, such as viral infection history prior to *M*. *ulcerans* infection to identify factors promoting the type I IFN response and potentially influencing disease severity in absence of antibiotic treatment [39,49]. Our work, implicating innate immune tolerance in the resolution of inflammation in *M*. *ulcerans* infection, suggests that modulation of type I IFN is a rational strategy for the improved treatment of Buruli ulcer to induce wound healing responses instead of rampant inflammation.

## Materials and methods

### Ethics statement for animal experiments and the use of human tissues

All animal experiments were performed in full compliance with national (articles R214-87 to R214-90 of the French “rural code”) and European (directive 2010/63/EU of the European Parliament and of the Council of September 22, 2010 on the protection of animals used for scientific purposes) guidelines. All protocols were approved by the Ethics Committee of the Pays de la Loire region, under protocol nos. CEEA 2009.14 and CEEA 2012.145. Animals were maintained under specific pathogen-free conditions in the animal housing facility of Angers University Hospital, France (agreement A 49 007 002). The use of biopsy samples from patients for research purposes was approved by the research committee of the government of Benin (Ministry of Health, Republic of Benin, agreement number 2893). The genetic studies of susceptibility to Buruli ulcer (BU) were approved by the institutional review board of the CDTLUB (Centre de Diagnostic et de Traitement de la Lèpre et de l’Ulcère de Buruli) and the national Buruli ulcer control authorities in Benin (IRB00006860), and by the ethics committee of Angers University Hospital, France. All participants provided written informed consent or had their parents provide written informed consent on their behalf.

### *M*. *ulcerans*, vesicles and mycolactone

*M*. *ulcerans* strain 1615 was originally isolated from patients in Malaysia. *M*. *ulcerans* 1615 and its mycolactone-deficient mutant (Tn:118 mutant) were used for the purification of mycolactone and MEVs. These strains were grown on solid 7H10 medium supplemented with 10% OADC (oleic acid, dextrose, catalase; Difco, Becton-Dickinson and Tween 80 0.05%, Sigma), as previously described [11]. After 35 days of culture, mycolactone or vesicles were purified from the pellet, as previously described [11]. Mycolactone was stored in absolute ethanol at -20°C in the dark, and vesicles were stored in PBS at -80°C.

### Mycolactone concentration and vesicle characterization

The amount of purified mycolactone, or of mycolactone present in the MEVs, was determined by high-performance liquid chromatography (HPLC) on a C18 column, as previously described [11]. *M*. *ulcerans* vesicles were characterized by nanoparticle tracking analysis (NTA) on the Nano-sight NS300 system (Malvern Instruments Ltd., Malvern, United Kingdom) equipped with an sCMOS camera and a blue laser (488 nm) to illuminate particles within the 10–2000 nm size range, as previously described [11].

### Footpad swelling

We injected PBS or MEVs-PM containing a total of 2 μg mycolactone into the footpads of 8-12-week-old female FVB/N mice (Janvier, Le Genest Saint Isle, France) and BALB/c mice (Charles River Laboratories, Saint-Germain-Nuelles, France). Three days later, mice were injected with MEVs-PM (2 μg mycolactone). We measured the footpad swelling using a caliper 24 hours following the last injection.

### Histology

The inoculated mice were sacrificed 24h following the last injection and the footpad was excised and immediately fixed by incubation in 4% paraformaldehyde (PFA) for 24 h. Tissues were then embedded in paraffin blocks, which were cut into 3 μm-thick sections. Hematoxylin-phloxine-saffron (HPS) staining was performed, according to the kit manufacturer’s protocol.

### Murine bone marrow-derived macrophages

Bone marrow-derived macrophages (BMDMs) were generated by culturing bone marrow pro-genitors (5 x 10^5^ cells/mL) from the femur bones of BALB/c and FVB/N mice for seven days in RPMI medium containing FBS (10%) and M-CSF (20 ng/mL) (Immunotools). Macrophages were used to seed 12-well plates at a density of 10^6^ cells/well and were incubated as indicated for each experiment at 37°C in 5% CO^2^. For ELISA analysis, supernatants were collected 24 hours following the last incubation. For RNA sequencing processing, RNA was collected at 6 hours after the last incubation and stored frozen at -80°C until mRNA extraction. Recombinant IFN-β (1 and 10 ng/mL) and IFN-β blocking antibodies (10 ug/mL) were purchased from Biolegends.

### Mouse infection experiment

A bacterial suspension of *M*. *ulcerans* was prepared, as previously described [11] and its concentration was adjusted to 2 x 10^5^ acid-fast bacilli/mL for an inoculum of 1 x 10^4^ bacilli in 50 μL, which was injected into the tail dermis of six-week-old female consanguineous BALB/c or FVB/N mice. Mice were euthanized when they reached the ulcerative stage of the infection (40 days), and the skin from tails were collected and stored frozen at -80°C until mRNA extraction.

### Human biopsies

We selected 14 patients (upon treatment at the Centre de diagnostic et de traitement de la lèpre et de l’ulcère de Buruli (CDTLUB) in Pobè). Biopsies were conserved in RNALater reagent (QIAGEN) at -20°C and total RNA were extracted using the RNeasy Fibrous Tissue Midi Kit (QIAGEN) according to the manufacturer’s protocol

### RT-qPCR

For mouse tissues, the inoculated mice were sacrificed 24h following the last injection and the footpad was excised and immediately frozen in liquid nitrogen. Footpads were grinded using liquid nitrogen and resuspended in TRIzol reagent (Invitrogen). For cells, TRIzol was directly added at the end of the experiment. TRIzol samples (from cells and footpads) were processed using the direct-zol RNA miniprep kit. Collected RNAs (Cells, footpads, human biopsies) were treated with DNase and transcribed into cDNA by using MLV reverse transcriptase (Invitrogen). We conducted qPCR using SYBR Green master mix (Thermofisher). Transcript levels of individual genes were measured using specific primers (m-*β-actin*-F: GGCTGTATTCCCCTCCATCG, R: CCAGTTGGTAACAATGCCATGT, m-*ifnb*-F: TCCCACGTCAATCTTTCCTGTTGCTTT, R: AACCTCACCTACAGGGCGGACTTCA, m-s*etdb2*-F: GGATGGAGCTACAAGATGATGG, R: CCAGTGTTTGCGTGTTACTCAG, h-*gapdh*-F: CTGGGCTACACTGAGCACC, R: AAGTGGTCGTTGAGGGCAATG, h-*il6*-F: ACTCACCTCTTCAGAACGAATTG, R: CCATCTTTGGAAGGTTCAGGTTG, h-*il1b*-F: TGAGCTCGCCAGTGAAATGA, R: AGGAGCACTTCATCTGTTTAGGG, h-*ifnb*-F: ATGACCAACAAGTGTCTCCTCC R: GGAATCCAAGCAAGTTGTAGCTC) and calculated using the 2−ΔCt method and presented as number of copies compared with control gene expression (*gapdh*) for human data or as fold change over the control condition (normalized with *β-actin*) for mouse data.

### ELISA and multiplex quantification

IL-1β, IL-6 and IFN-β were quantified with a quantitative ELISA kit, according to the manufacturer’s instructions (Invitrogen, Thermo Fisher Scientific). The levels of mouse CXCL1, CCL3, IL1α, IL1β TNFα, IL6, CCL2, IL33, CXCL2, IL10, TMP-1 and VEGF were determined with a Luminex assay kit (R&D Systems).

### RNA sequencing and analysis

Tissues were grinded with beads using a beat beater (Mixer Mill MM301, Retsch GmbH). RNAs from grinded tissues or collected cells were purified using direct-zol RNA mini prep kit (Zymo research). RNA pellets were sent to BGI for RNA sequencing and were treated as follows: oligo(dT)-conjugated magnetic beads were used to purified mRNA. The purified mRNA was then fragmented into small pieces in fragmentation buffer at the appropriate temperature. The first-strand cDNA was then generated by reverse transcription with random hexamer primers, and the second strand was generated with a strand synthesis reaction system (using dUTP instead of dTTP). The reaction product was purified with magnetic beads, and A-Tailing Mix and RNA index adapters were added, with the mixture incubated until end repair was complete. The cDNA fragments with adapters thus obtained were amplified by several rounds of PCR, and the U-labeled second-strand template was digested with UDG enzyme. PCR products were purified with XP beads and dissolved in EB solution. The quality of the library was assessed with two methods. First, we checked the distribution of fragment size with an Agilent 2100 bioanalyzer. The double-stranded PCR products were denatured by heating and circularized by the splint-oligo sequence. The single-strand circlular DNAs (ssCir DNA) were formatted to generate the final library. The library was amplified with phi29 to obtain a DNA nanoball (DNB), with more than 300 copies of each molecule. The DNBs were loaded into the patterned nanoarray and paired-end 100-base reads were sequenced by combinatorial Probe-Anchor Synthesis (cPAS) on a DNBseq sequencer. The sample and group comparison were made according to the poisson distribution and the DESeq2 method respectively. The sequencing analysis was performed, and graph (heatmap/pathway enrichment) were generated with Dr. Tom software (BGI).

## Supporting information

S1 Fig Quantification of toxicity and IL-6 secretion in a dose-response of mycolactone on FVB/N and Balb/c macrophages. **A.** BMDM cells were stimulated with mycolactone at dose of 3, 6 or 12 or 24 ng/ml during 48h. Cytotoxic effect was recorded using ToxiLight bioassay kit (Lonza). **B.** IL-6 were detected in supernatant of cells by ELISA at 48h post-stimulation with NPM.
(DOCX)

S2 Fig BALB/c and FVB/N fibroblasts develop a tolerant phenotype following repeated exposures to *M*. *ulcerans* antigens whether or not mycolactone is present. Fibroblasts were isolated from BALB/c and FVB/N mouse tails and cultured for three passages. Cells were seeded into plates and incubated for 24 hours +/- vesicles from a mycolactone deficient strain (MEVs NPM, MOI: 20,000) +/- purified mycolactone (Myco, 6 ng/mL). Cells were washed with PBS and activated with the same ligands for another 24 hours. Supernatants were collected. IL-6 secretion was measured by ELISA. Bars represent the mean ± SEM. Data are representative of 4 independent experiments. Statistical analysis was performed using one-way ANOVA with Tukey post-test (**P < 0.005, ***p<0.001).
(DOCX)

S3 Fig Summary of differentially regulated genes in BALB/c and FVB/N macrophages following repeated exposure to M. ulcerans vesicles and mycolactone. (A) Heatmap of 87 genes downregulated in BALB/c macrophages only, highlighted in Fig 3B. (B) Pathway enrichment of downregulated genes in BALB/c macrophages only. (C) Heatmap of 58 genes upregulated in FVB/N macrophages only, highlighted in Fig 3E. (D) Pathway enrichment of upregulated genes in FVB/N macrophages only. For (B) and (D), the Rich ratio is the ratio of the number of differentially expressed genes annotated in this pathway relative to all genes annotated in this pathway. A Q value is the corrected p value ranging from 0 to 1. Q values < 0.05 are considered significant. Data are based on three independent replicates.
(DOCX)

S4 Fig BALB/c but not FVB/N macrophages fail to develop immune tolerance against *M*. *ulcerans* vesicles in the presence of mycolactone at the mRNA level. Macrophages were seeded into plates and incubated for 24 hours +/- vesicles from a mycolactone deficient strain (MEVs NPM, MOI: 20,000) +/- purified mycolactone (Myco, 6 ng/mL). Cells were washed with PBS and activated with the same ligands for six hours and mRNA was collected. Gene expression of cytokine genes (*cxcl1*, *il6*, *ccl3*, *tnfa*, *il1a* and *il1b*) was measured by RNAseq. Bars represent the mean ± SEM. Data are based on three independent replicates. Statistical analysis was performed using one-way ANOVA with Tukey post-test (*NS* = not significant, *P < 0.05, **P < 0.005, ***p<0.001).
(DOCX)

S5 Fig Genes induced during the first exposure to *M*. *ulcerans* vesicles with purified mycolactone in BALB/c macrophages. Macrophages from BALB/c and FVB/N mice were seeded into plates and incubated for six hours +/- vesicles from a mycolactone deficient strain (MEVs NPM, MOI: 20,000) +/- purified mycolactone (Myco, 6 ng/mL). mRNA was collected and gene expression was measured by RNAseq. (A) Differential gene expression summary comparing cells stimulated with MEVs NPM + Myco vs. cells stimulated only MEVs NPM only, for each mouse strain. Genes induced have log2FC > = 2, Q value < = 0.05. (B) Heatmap of 9 genes induced in BALB/c macrophages only highlighted in Fig 4A. Data are based on three independent replicates.
(DOCX)

S6 Fig Genes induced during infection with *M*. *ulcerans* in BALB/c mice only. BALB/c and FVB/N mice were injected in the tail with living *M*. *ulcerans* organisms. Mice were sacrificed at the ulcerative stage and the skin from tails was collected. mRNA was collected and gene expression was measured by RNAseq. (A) Heatmap of the genes induced in infected BALB/c mice only as compared to control (log2FC > = 1.5, Q value < = 0.05). (B) Pathway enrichment of genes induced only in infected BALB/c mice. the Rich ratio is the ratio of the number of differentially expressed genes annotated in this pathway relative to all genes annotated in this pathway. A Q value is the corrected p value ranging from 0 to 1. Q values < 0.05 are considered significant.
(DOCX)

S1 Data Differentially regulated genes in BALB/c and FVB/N macrophages following one or two exposure to *M*. *ulcerans* vesicles and mycolactone. The differentially regulated genes in figure 3, 4, 5 and 6 are available in this file.
(XLSX)

S1 Table Duration of antibiotic treatment before human tissue collection. (DOCX)

## References

[1] Simpson H, Deribe K, Tabah EN, Peters A, Maman I, Frimpong M, et al. Mapping the global distribution of Buruli ulcer: a systematic review with evidence consensus. Lancet Glob Health. 2019;7(7):e912–e22. doi: 10.1016/S2214-109X(19)30171-8 31200890 PMC6614043

[2] Guarner J, Bartlett J, Whitney EA, Raghunathan PL, Stienstra Y, Asamoa K, et al. Histopathologic features of Mycobacterium ulcerans infection. Emerg Infect Dis. 2003;9(6):651–6. doi: 10.3201/eid0906.020485 12780997 PMC3000137

[3] George KM, Chatterjee D, Gunawardana G, Welty D, Hayman J, Lee R, et al. Mycolactone: a polyketide toxin from Mycobacterium ulcerans required for virulence. Science. 1999;283(5403):854–7. doi: 10.1126/science.283.5403.854 9933171

[4] Marsollier L, Aubry J, Coutanceau E, Andre JP, Small PL, Milon G, et al. Colonization of the salivary glands of Naucoris cimicoides by Mycobacterium ulcerans requires host plasmatocytes and a macrolide toxin, mycolactone. Cell Microbiol. 2005;7(7):935–43. doi: 10.1111/j.1462-5822.2005.00521.x 15953026

[5] Strong E, Hart B, Wang J, Orozco MG, Lee S. Induced Synthesis of Mycolactone Restores the Pathogenesis of Mycobacterium ulcerans In Vitro and In Vivo. Front Immunol. 2022;13:750643. doi: 10.3389/fimmu.2022.750643 35401531 PMC8988146

[6] Hall BS, Hill K, McKenna M, Ogbechi J, High S, Willis AE, et al. The pathogenic mechanism of the Mycobacterium ulcerans virulence factor, mycolactone, depends on blockade of protein translocation into the ER. PLoS Pathog. 2014;10(4):e1004061. doi: 10.1371/journal.ppat.1004061 24699819 PMC3974873

[7] Ogbechi J, Hall BS, Sbarrato T, Taunton J, Willis AE, Wek RC, et al. Inhibition of Sec61-dependent translocation by mycolactone uncouples the integrated stress response from ER stress, driving cytotoxicity via translational activation of ATF4. Cell Death Dis. 2018;9(3):397. doi: 10.1038/s41419-018-0427-y 29540678 PMC5852046

[8] Baron L, Paatero AO, Morel JD, Impens F, Guenin-Mace L, Saint-Auret S, et al. Mycolactone subverts immunity by selectively blocking the Sec61 translocon. J Exp Med. 2016;213(13):2885–96. doi: 10.1084/jem.20160662 27821549 PMC5154940

[9] Demangel C, High S. Sec61 blockade by mycolactone: A central mechanism in Buruli ulcer disease. Biol Cell. 2018;110(11):237–48. doi: 10.1111/boc.201800030 30055020

[10] Hall B, Simmonds R. Pleiotropic molecular effects of the Mycobacterium ulcerans virulence factor mycolactone underlying the cell death and immunosuppression seen in Buruli ulcer. Biochem Soc Trans. 2014;42(1):177–83. doi: 10.1042/BST20130133 24450648

[11] Foulon M, Robbe-Saule M, Manry J, Esnault L, Boucaud Y, Alcais A, et al. Mycolactone toxin induces an inflammatory response by targeting the IL-1beta pathway: Mechanistic insight into Buruli ulcer pathophysiology. PLoS Pathog. 2020;16(12):e1009107. 33338061 10.1371/journal.ppat.1009107 PMC7748131

[12] Hall BS, Hsieh LT, Sacre S, Simmonds RE. The One That Got Away: How Macrophage-Derived IL-1beta Escapes the Mycolactone-Dependent Sec61 Blockade in Buruli Ulcer. Front Immunol. 2021;12:788146. 35154073 10.3389/fimmu.2021.788146 PMC8826060

[13] Gupta S, Rodriguez GM. Mycobacterial extracellular vesicles and host pathogen interactions. Pathog Dis. 2018;76(4). doi: 10.1093/femspd/fty031 29722822 PMC5930244

[14] Marsollier L, Brodin P, Jackson M, Kordulakova J, Tafelmeyer P, Carbonnelle E, et al. Impact of Mycobacterium ulcerans biofilm on transmissibility to ecological niches and Buruli ulcer pathogenesis. PLoS Pathog. 2007;3(5):e62. doi: 10.1371/journal.ppat.0030062 17480118 PMC1864991

[15] Marion E, Chauty A, Kempf M, Le Corre Y, Delneste Y, Croue A, et al. Clinical Features of Spontaneous Partial Healing During Mycobacterium ulcerans Infection. Open Forum Infect Dis. 2016;3(1):ofw013. doi: 10.1093/ofid/ofw013 26925431 PMC4767261

[16] Portaels F, Silva MT, Meyers WM. Buruli ulcer. Clin Dermatol. 2009;27(3):291–305. doi: 10.1016/j.clindermatol.2008.09.021 19362692

[17] Netea MG, Dominguez-Andres J, Barreiro LB, Chavakis T, Divangahi M, Fuchs E, et al. Defining trained immunity and its role in health and disease. Nat Rev Immunol. 2020;20(6):375–88. doi: 10.1038/s41577-020-0285-6 32132681 PMC7186935

[18] Sherwood ER, Burelbach KR, McBride MA, Stothers CL, Owen AM, Hernandez A, et al. Innate Immune Memory and the Host Response to Infection. J Immunol. 2022;208(4):785–92. doi: 10.4049/jimmunol.2101058 35115374 PMC8982914

[19] Krahenbuhl JL, Sharma SD, Ferraresi RW, Remington JS. Effects of muramyl dipeptide treatment on resistance to infection with Toxoplasma gondii in mice. Infect Immun. 1981;31(2):716–22. doi: 10.1128/iai.31.2.716-722.1981 7216470 PMC351369

[20] Teufel LU, Joosten LAB, Dos Santos JC. Immunotherapeutic Potential of Interleukin-32 and Trained Immunity for Leishmaniasis Treatment. Trends Parasitol. 2021;37(2):130–41. doi: 10.1016/j.pt.2020.09.014 33082090

[21] Verrall AJ, Schneider M, Alisjahbana B, Apriani L, van Laarhoven A, Koeken V, et al. Early Clearance of Mycobacterium tuberculosis Is Associated With Increased Innate Immune Responses. J Infect Dis. 2020;221(8):1342–50. doi: 10.1093/infdis/jiz147 30958547

[22] Guillon A, Arafa EI, Barker KA, Belkina AC, Martin I, Shenoy AT, et al. Pneumonia recovery reprograms the alveolar macrophage pool. JCI Insight. 2020;5(4). doi: 10.1172/jci.insight.133042 31990682 PMC7101156

[23] Schrum JE, Crabtree JN, Dobbs KR, Kiritsy MC, Reed GW, Gazzinelli RT, et al. Cutting Edge: Plasmodium falciparum Induces Trained Innate Immunity. J Immunol. 2018;200(4):1243–8. doi: 10.4049/jimmunol.1701010 29330325 PMC5927587

[24] Bernard Q, Hu LT. Innate Immune Memory to Repeated Borrelia burgdorferi Exposure Correlates with Murine In Vivo Inflammatory Phenotypes. J Immunol. 2020;205(12):3383–9. doi: 10.4049/jimmunol.2000686 33168577 PMC7725865

[25] Riksen NP. Trained immunity and atherosclerotic cardiovascular disease. Curr Opin Lipidol. 2019;30(5):395–400. doi: 10.1097/MOL.0000000000000628 31335332

[26] Cavaillon JM, Singer M, Skirecki T. Sepsis therapies: learning from 30 years of failure of translational research to propose new leads. EMBO Mol Med. 2020;12(4):e10128. doi: 10.15252/emmm.201810128 32176432 PMC7136965

[27] Watson DW, Kim YB. Modification of Host Responses to Bacterial Endotoxins. I. Specificity of Pyrogenic Tolerance and the Role of Hypersensitivity in Pyrogenicity, Lethality, and Skin Reactivity. J Exp Med. 1963;118(3):425–46. doi: 10.1084/jem.118.3.425 14078002 PMC2137653

[28] Funes SC, Rios M, Fernandez-Fierro A, Di Genaro MS, Kalergis AM. Trained Immunity Contribution to Autoimmune and Inflammatory Disorders. Front Immunol. 2022;13:868343. doi: 10.3389/fimmu.2022.868343 35464438 PMC9028757

[29] Jiao Y, Wu L, Huntington ND, Zhang X. Crosstalk Between Gut Microbiota and Innate Immunity and Its Implication in Autoimmune Diseases. Front Immunol. 2020;11:282. doi: 10.3389/fimmu.2020.00282 32153586 PMC7047319

[30] Nakatsuji T, Cheng JY, Gallo RL. Mechanisms for control of skin immune function by the microbiome. Curr Opin Immunol. 2021;72:324–30. doi: 10.1016/j.coi.2021.09.001 34537476

[31] Bolz M, Ruf MT. Buruli Ulcer in Animals and Experimental Infection Models. In: Pluschke G, Roltgen K, editors. Buruli Ulcer: Mycobacterium Ulcerans Disease. Cham (CH)2019. p. 159–81. 32091701

[32] Johnson PD, Stinear T, Small PL, Pluschke G, Merritt RW, Portaels F, et al. Buruli ulcer (M. ulcerans infection): new insights, new hope for disease control. PLoS Med. 2005;2(4):e108. doi: 10.1371/journal.pmed.0020108 15839744 PMC1087202

[33] Marion E, Jarry U, Cano C, Savary C, Beauvillain C, Robbe-Saule M, et al. FVB/N Mice Spontaneously Heal Ulcerative Lesions Induced by Mycobacterium ulcerans and Switch M. ulcerans into a Low Mycolactone Producer. J Immunol. 2016;196(6):2690–8. doi: 10.4049/jimmunol.1502194 26873988

[34] Hall BS, Dos Santos SJ, Hsieh LT, Manifava M, Ruf MT, Pluschke G, et al. Inhibition of the SEC61 translocon by mycolactone induces a protective autophagic response controlled by EIF2S1-dependent translation that does not require ULK1 activity. Autophagy. 2021:1–19. 10.1080/15548627.2021.1961067 PMC9037441 34424124

[35] Beeson PB. Development of tolerance to typhoid bacterial pyrogen and its abolition by reticulo-endothelial blockade. Proc Soc Exp Biol Med. 1946;61:248–50. doi: 10.3181/00379727-61-15291p 21024160

[36] Seeley JJ, Ghosh S. Molecular mechanisms of innate memory and tolerance to LPS. J Leukoc Biol. 2017;101(1):107–19. doi: 10.1189/jlb.3MR0316-118RR 27780875

[37] Benveniste EN, Qin H. Type I interferons as anti-inflammatory mediators. Sci STKE. 2007;2007(416):pe70. doi: 10.1126/stke.4162007pe70 18073382

[38] Stack J, Doyle SL, Connolly DJ, Reinert LS, O’Keeffe KM, McLoughlin RM, et al. TRAM is required for TLR2 endosomal signaling to type I IFN induction. J Immunol. 2014;193(12):6090–102. doi: 10.4049/jimmunol.1401605 25385819 PMC4258402

[39] Schliehe C, Flynn EK, Vilagos B, Richson U, Swaminanthan S, Bosnjak B, et al. The methyltransferase Setdb2 mediates virus-induced susceptibility to bacterial superinfection. Nat Immunol. 2015;16(1):67–74. doi: 10.1038/ni.3046 25419628 PMC4320687

[40] Romee R, Rosario M, Berrien-Elliott MM, Wagner JA, Jewell BA, Schappe T, et al. Cytokine-induced memory-like natural killer cells exhibit enhanced responses against myeloid leukemia. Sci Transl Med. 2016;8(357):357ra123. doi: 10.1126/scitranslmed.aaf2341 27655849 PMC5436500

[41] Mayer-Barber KD, Yan B. Clash of the Cytokine Titans: counter-regulation of interleukin-1 and type I interferon-mediated inflammatory responses. Cell Mol Immunol. 2017;14(1):22–35. doi: 10.1038/cmi.2016.25 27264686 PMC5214938

[42] Ashley CL, Abendroth A, McSharry BP, Slobedman B. Interferon-Independent Upregulation of Interferon-Stimulated Genes during Human Cytomegalovirus Infection is Dependent on IRF3 Expression. Viruses. 2019;11(3). doi: 10.3390/v11030246 30871003 PMC6466086

[43] John SP, Sun J, Carlson RJ, Cao B, Bradfield CJ, Song J, et al. IFIT1 Exerts Opposing Regulatory Effects on the Inflammatory and Interferon Gene Programs in LPS-Activated Human Macrophages. Cell Rep. 2018;25(1):95–106 e6. doi: 10.1016/j.celrep.2018.09.002 30282041 PMC6492923

[44] Anzaghe M, Kronhart S, Niles MA, Hocker L, Dominguez M, Kochs G, et al. Type I interferon receptor-independent interferon-alpha induction upon infection with a variety of negative-strand RNA viruses. J Gen Virol. 2021;102(7). 10.1099/jgv.0.001616 34269676

[45] Isaacs A, Lindenmann J. Virus interference. I. The interferon. Proc R Soc Lond B Biol Sci. 1957;147(927):258–67. doi: 10.1098/rspb.1957.0048 13465720

[46] Schneider WM, Chevillotte MD, Rice CM. Interferon-stimulated genes: a complex web of host defenses. Annu Rev Immunol. 2014;32:513–45. doi: 10.1146/annurev-immunol-032713-120231 24555472 PMC4313732

[47] Decker T, Muller M, Stockinger S. The yin and yang of type I interferon activity in bacterial infection. Nat Rev Immunol. 2005;5(9):675–87. doi: 10.1038/nri1684 16110316

[48] Hedges JF, Robison A, Kimmel E, Christensen K, Lucas E, Ramstead A, et al. Type I Interferon Counters or Promotes Coxiella burnetii Replication Dependent on Tissue. Infect Immun. 2016;84(6):1815–25. doi: 10.1128/IAI.01540-15 27068091 PMC4907146

[49] Li W, Moltedo B, Moran TM. Type I interferon induction during influenza virus infection increases susceptibility to secondary Streptococcus pneumoniae infection by negative regulation of gammadelta T cells. J Virol. 2012;86(22):12304–12. 22951826 10.1128/JVI.01269-12 PMC3486468

[50] LeMessurier KS, Hacker H, Chi L, Tuomanen E, Redecke V. Type I interferon protects against pneumococcal invasive disease by inhibiting bacterial transmigration across the lung. PLoS Pathog. 2013;9(11):e1003727. doi: 10.1371/journal.ppat.1003727 24244159 PMC3820719

[51] Georgel P. Crosstalk between Interleukin-1beta and Type I Interferons Signaling in Autoinflammatory Diseases. Cells. 2021;10(5). 10.3390/cells10051134 PMC8150590 34066649

[52] Guarda G, Braun M, Staehli F, Tardivel A, Mattmann C, Forster I, et al. Type I interferon inhibits interleukin-1 production and inflammasome activation. Immunity. 2011;34(2):213–23. doi: 10.1016/j.immuni.2011.02.006 21349431

[53] Novikov A, Cardone M, Thompson R, Shenderov K, Kirschman KD, Mayer-Barber KD, et al. Mycobacterium tuberculosis triggers host type I IFN signaling to regulate IL-1beta production in human macrophages. J Immunol. 2011;187(5):2540–7. 21784976 10.4049/jimmunol.1100926 PMC3159798

[54] Kumaran Satyanarayanan S, El Kebir D, Soboh S, Butenko S, Sekheri M, Saadi J, et al. IFN-beta is a macrophage-derived effector cytokine facilitating the resolution of bacterial inflammation. Nat Commun. 2019;10(1):3471. 31375662 10.1038/s41467-019-10903-9 PMC6677895

[55] Koh TJ, DiPietro LA. Inflammation and wound healing: the role of the macrophage. Expert Rev Mol Med. 2011;13:e23. doi: 10.1017/S1462399411001943 21740602 PMC3596046

[56] Kimball AS, Davis FM, denDekker A, Joshi AD, Schaller MA, Bermick J, et al. The Histone Methyltransferase Setdb2 Modulates Macrophage Phenotype and Uric Acid Production in Diabetic Wound Repair. Immunity. 2019;51(2):258–71 e5. doi: 10.1016/j.immuni.2019.06.015 31350176 PMC6703945

[57] El Gazzar M, Yoza BK, Hu JY, Cousart SL, McCall CE. Epigenetic silencing of tumor necrosis factor alpha during endotoxin tolerance. J Biol Chem. 2007;282(37):26857–64. doi: 10.1074/jbc.M704584200 17646159

[58] Vincent QB, Belkadi A, Fayard C, Marion E, Adeye A, Ardant MF, et al. Microdeletion on chromosome 8p23.1 in a familial form of severe Buruli ulcer. PLoS Negl Trop Dis. 2018;12(4):e0006429. doi: 10.1371/journal.pntd.0006429 29708969 PMC5945055

[59] Capela C, Dossou AD, Silva-Gomes R, Sopoh GE, Makoutode M, Menino JF, et al. Genetic Variation in Autophagy-Related Genes Influences the Risk and Phenotype of Buruli Ulcer. PLoS Negl Trop Dis. 2016;10(4):e0004671. doi: 10.1371/journal.pntd.0004671 27128681 PMC4851401

[60] Martin PK, Marchiando A, Xu R, Rudensky E, Yeung F, Schuster SL, et al. Autophagy proteins suppress protective type I interferon signalling in response to the murine gut microbiota. Nat Microbiol. 2018;3(10):1131–41. doi: 10.1038/s41564-018-0229-0 30202015 PMC6179362

