# Supplementary material for: Type-I interferons promote innate immune tolerance in macrophages exposed to Mycobacterium ulcerans vesicles
Source: PLoS Pathog. 2023 Jul 10;19(7):e1011479. doi: 10.1371/journal.ppat.1011479 (PMC10358927; doi:10.1371/journal.ppat.1011479)
Supplement: S1 Fig — A. BMDM cells were stimulated with mycolactone at dose of 3, 6 or 12 or 24 ng/ml during 48h. Cytotoxic effect was recorded using ToxiLight bioassay kit (Lonza). B. IL-6 were detected in supernatant of cells by ELISA at 48h post-stimulation with NPM. (DOCX) [file ppat.1011479.s001.docx]

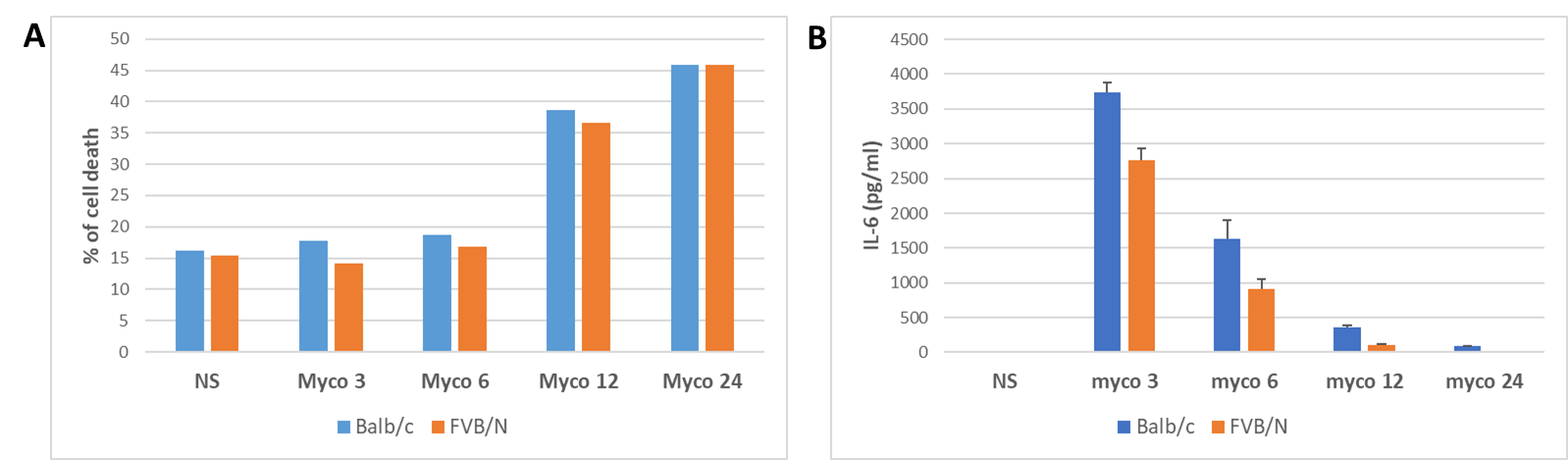


**Figure S1 : Quantification of toxicity and IL-6 secretion in a dose-response of mycolactone on FVB/N and Balb/c macrophages.**

**A.** BMDM cells were stimulated with mycolactone at dose of 3, 6 or 12 or 24 ng/ml during 48h. Cytotoxic effect was recorded using ToxiLight bioassay kit (Lonza). **B.** IL-6 were detected in supernatant of cells by ELISA at 48h post-stimulation with NPM.
