# Supplementary material for: Type-I interferons promote innate immune tolerance in macrophages exposed to Mycobacterium ulcerans vesicles
Source: PLoS Pathog. 2023 Jul 10;19(7):e1011479. doi: 10.1371/journal.ppat.1011479 (PMC10358927; doi:10.1371/journal.ppat.1011479)
Supplement: S2 Fig — Fibroblasts were isolated from BALB/c and FVB/N mouse tails and cultured for three passages. Cells were seeded into plates and incubated for 24 hours +/- vesicles from a mycolactone deficient strain (MEVs NPM, MOI: 20,000) +/- purified mycolactone (Myco, 6 ng/mL). Cells were washed with PBS and activated with the same ligands for another 24 hours. Supernatants were collected. IL-6 secretion was measured by ELISA. Bars represent the mean ± SEM. Data are representative of 4 independent experiments. Statistical analysis was performed using one-way ANOVA with Tukey post-test (**P < 0.005, ***p<0.001). (DOCX) [file ppat.1011479.s002.docx]

**Figure S2**. **BALB/c and FVB/N fibroblasts develop a tolerant phenotype following repeated exposures to *M. ulcerans* antigens whether or not mycolactone is present**. Fibroblasts were isolated from BALB/c and FVB/N mouse tails and cultured for three passages. Cells were seeded into plates and incubated for 24 hours +/- vesicles from a mycolactone deficient strain (MEVs NPM, MOI: 20,000) +/- purified mycolactone (Myco, 6 ng/mL). Cells were washed with PBS and activated with the same ligands for another 24 hours. Supernatants were collected. IL-6 secretion was measured by ELISA. Bars represent the mean ± SEM. Data are representative of 4 independent experiments. Statistical analysis was performed using one-way ANOVA with Tukey post-test (**P < 0.005, ***p<0.001).
