# Supplementary material for: Type-I interferons promote innate immune tolerance in macrophages exposed to Mycobacterium ulcerans vesicles
Source: PLoS Pathog. 2023 Jul 10;19(7):e1011479. doi: 10.1371/journal.ppat.1011479 (PMC10358927; doi:10.1371/journal.ppat.1011479)
Supplement: S3 Fig — (A) Heatmap of 87 genes downregulated in BALB/c macrophages only, highlighted in Fig 3B. (B) Pathway enrichment of downregulated genes in BALB/c macrophages only. (C) Heatmap of 58 genes upregulated in FVB/N macrophages only, highlighted in Fig 3E. (D) Pathway enrichment of upregulated genes in FVB/N macrophages only. For (B) and (D), the Rich ratio is the ratio of the number of differentially expressed genes annotated in this pathway relative to all genes annotated in this pathway. A Q value is the corrected p value ranging from 0 to 1. Q values < 0.05 are considered significant. Data are based on three independent replicates. (DOCX) [file ppat.1011479.s003.docx]

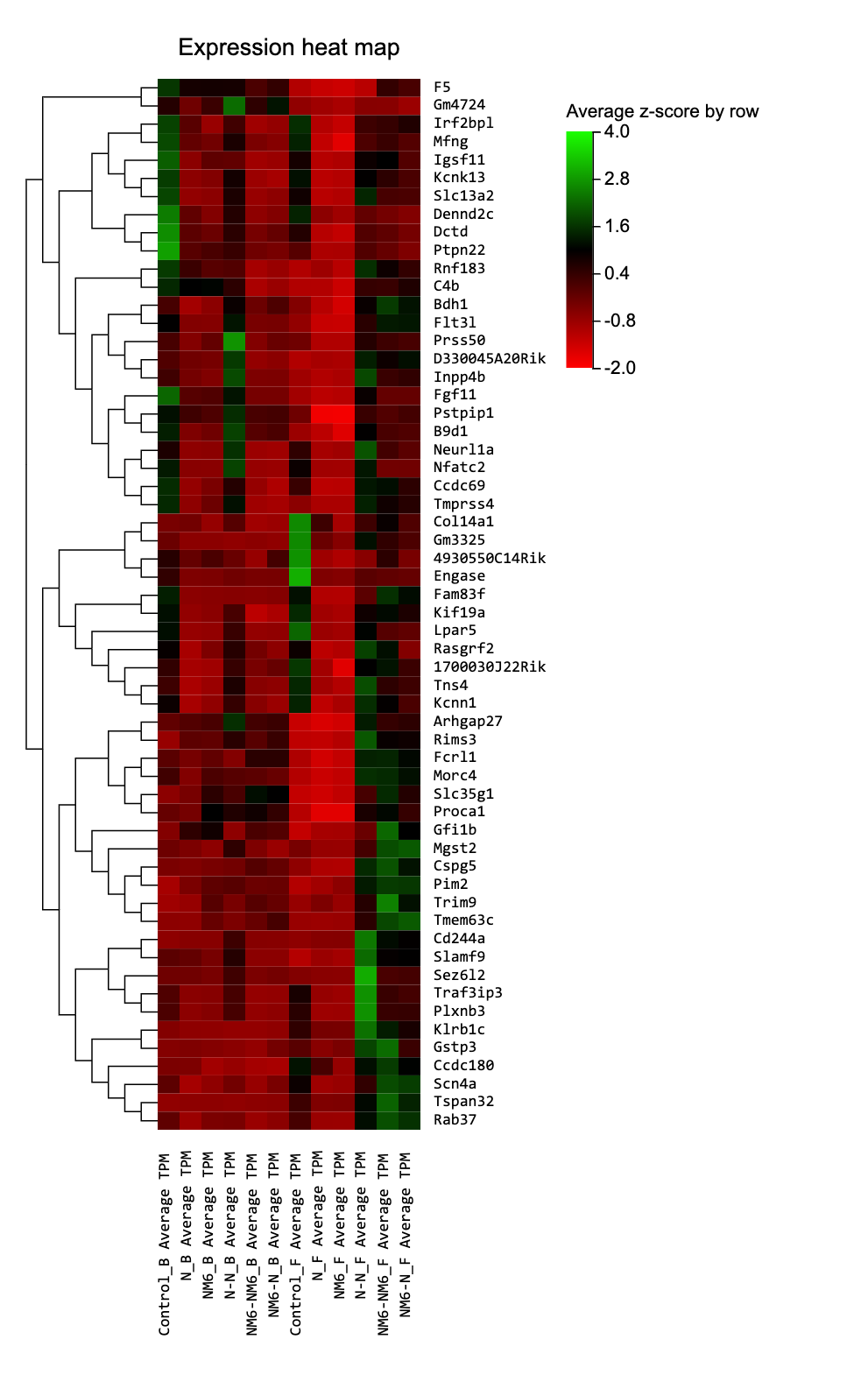


Average z-score by row

**All genes upregulated in FVB/N cells only**

1^st^

2^nd^

MFVs

MFVs

MEVs NPM

Myco

MEVs NPM

Myco


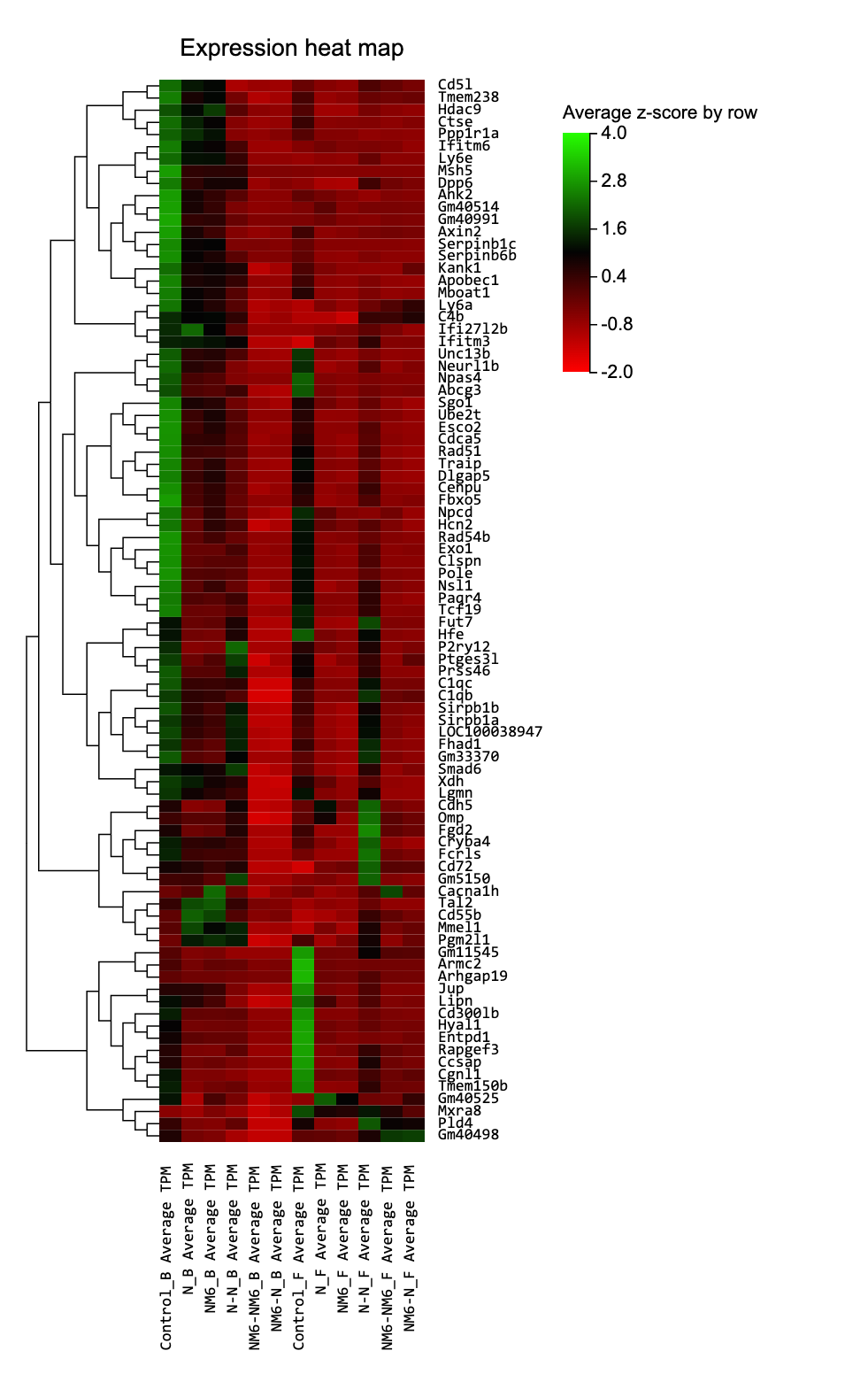


**All genes downregulated in BALB/c cells only**

Average z-score by row

1^st^

2^nd^

MFVs

MFVs

MEVs NPM

Myco

MEVs NPM

Myco


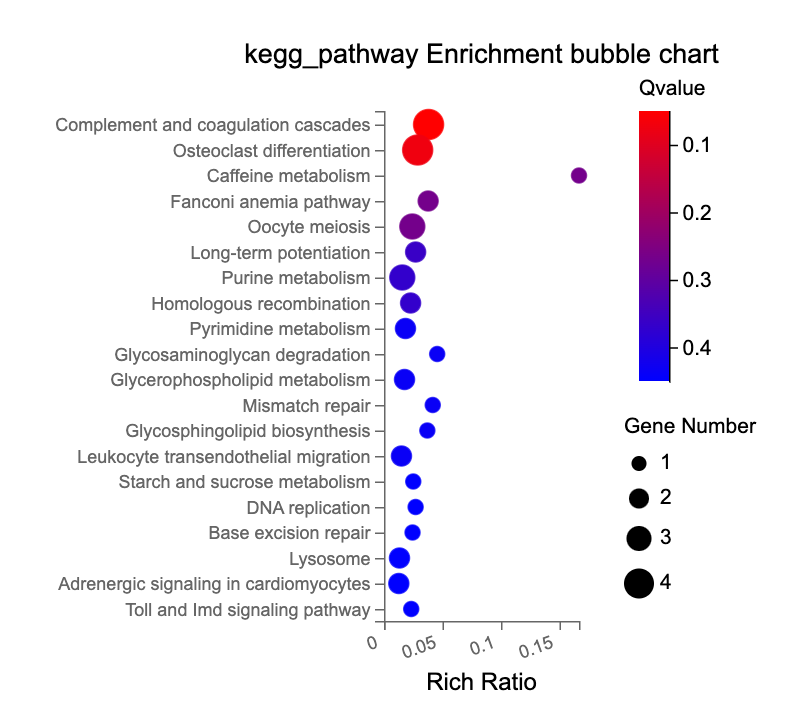


q>0.05

**B**

**Pathway enrichment of genes downregulated in BALB/c cells only**


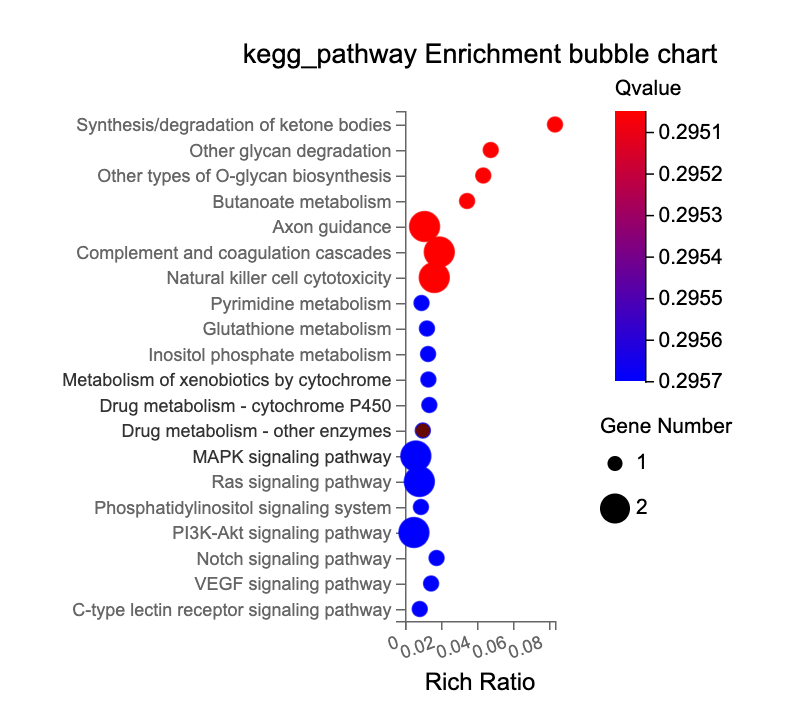


q>0.05

**D**

**Pathway enrichment of genes upregulated in FVB/N cells only**

**A**

**C**

- - - + + + - - - + + +

- - - - + + - - - - + +

- + + + + + - + + + + +

- - - + + + - - - + + +

- - - - + + - - - - + +

- + + + + + - + + + + +

FVB/N

BALB/c

FVB/N

BALB/c

- - + - - + - - + - - +

- - + - - + - - + - - +

**Figure S3. Summary of differentially regulated genes in BALB/c and FVB/N macrophages following repeated exposure to M. ulcerans vesicles and mycolactone.** (A) Heatmap of 87 genes downregulated in BALB/c macrophages only, highlighted in Fig. 3B. (B) Pathway enrichment of downregulated genes in BALB/c macrophages only. (C) Heatmap of 58 genes upregulated in FVB/N macrophages only, highlighted in Fig. 3E. (D) Pathway enrichment of upregulated genes in FVB/N macrophages only. For (B) and (D), the Rich ratio is the ratio of the number of differentially expressed genes annotated in this pathway relative to all genes annotated in this pathway. A Q value is the corrected p value ranging from 0 to 1. Q values < 0.05 are considered significant. Data are based on three independent replicates.
