# Supplementary material for: Type-I interferons promote innate immune tolerance in macrophages exposed to Mycobacterium ulcerans vesicles
Source: PLoS Pathog. 2023 Jul 10;19(7):e1011479. doi: 10.1371/journal.ppat.1011479 (PMC10358927; doi:10.1371/journal.ppat.1011479)
Supplement: S4 Fig — Macrophages were seeded into plates and incubated for 24 hours +/- vesicles from a mycolactone deficient strain (MEVs NPM, MOI: 20,000) +/- purified mycolactone (Myco, 6 ng/mL). Cells were washed with PBS and activated with the same ligands for six hours and mRNA was collected. Gene expression of cytokine genes (cxcl1, il6, ccl3, tnfa, il1a and il1b) was measured by RNAseq. Bars represent the mean ± SEM. Data are based on three independent replicates. Statistical analysis was performed using one-way ANOVA with Tukey post-test (NS = not significant, *P < 0.05, **P < 0.005, ***p<0.001). (DOCX) [file ppat.1011479.s004.docx]

**Figure S4. BALB/c but not FVB/N macrophages fail to develop immune tolerance against *M. ulcerans* vesicles in the presence of mycolactone at the mRNA level.** Macrophages were seeded into plates and incubated for 24 hours +/- vesicles from a mycolactone deficient strain (MEVs NPM, MOI: 20,000) +/- purified mycolactone (Myco, 6 ng/mL). Cells were washed with PBS and activated with the same ligands for six hours and mRNA was collected. Gene expression of cytokine genes (*cxcl1*, *il6*, *ccl3*, *tnfa*, *il1a* and *il1b*) was measured by RNAseq. Bars represent the mean ± SEM. Data are based on three independent replicates. Statistical analysis was performed using one-way ANOVA with Tukey post-test (*NS* = not significant, *P < 0.05, **P < 0.005, ***p<0.001).
