# Supplementary material for: Type-I interferons promote innate immune tolerance in macrophages exposed to Mycobacterium ulcerans vesicles
Source: PLoS Pathog. 2023 Jul 10;19(7):e1011479. doi: 10.1371/journal.ppat.1011479 (PMC10358927; doi:10.1371/journal.ppat.1011479)
Supplement: S5 Fig — Macrophages from BALB/c and FVB/N mice were seeded into plates and incubated for six hours +/- vesicles from a mycolactone deficient strain (MEVs NPM, MOI: 20,000) +/- purified mycolactone (Myco, 6 ng/mL). mRNA was collected and gene expression was measured by RNAseq. (A) Differential gene expression summary comparing cells stimulated with MEVs NPM + Myco vs. cells stimulated only MEVs NPM only, for each mouse strain. Genes induced have log2FC > = 2, Q value < = 0.05. (B) Heatmap of 9 genes induced in BALB/c macrophages only highlighted in Fig 4A. Data are based on three independent replicates. (DOCX) [file ppat.1011479.s005.docx]

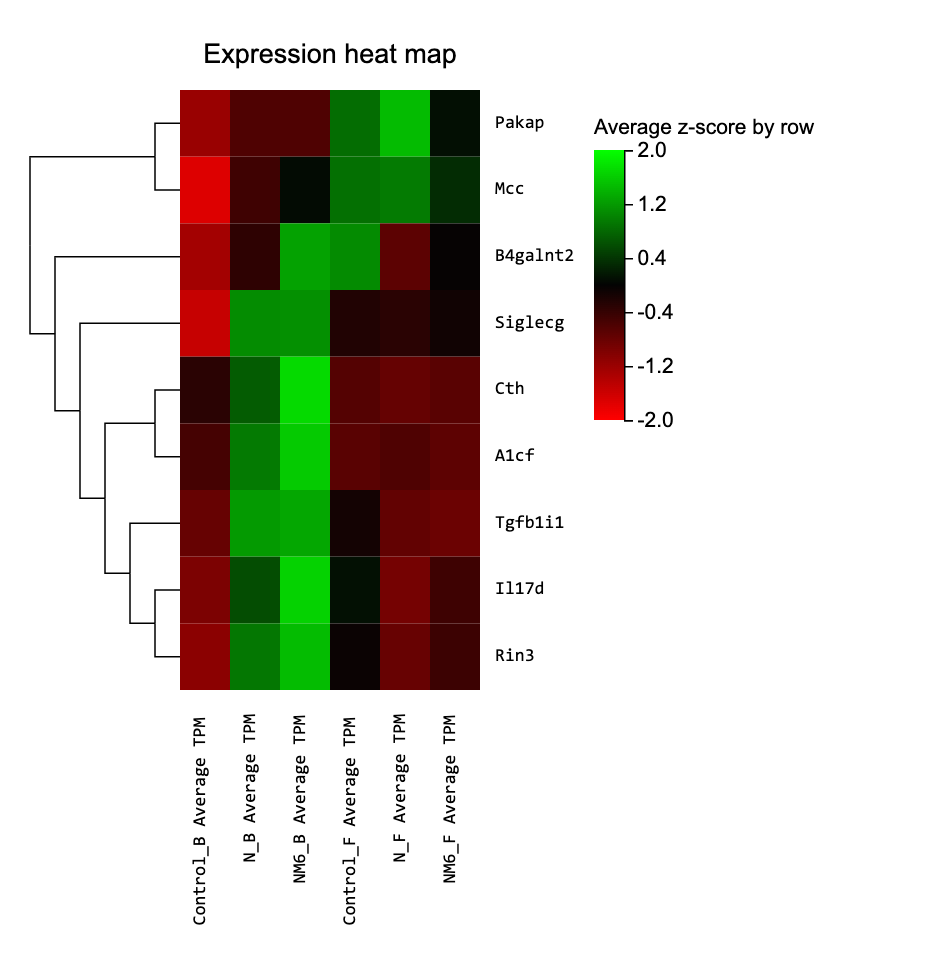


**Genes strongly induced in BALB/c cells only**

**B**

1 0 2

**Number of genes differentially expressed with versus without mycolactone**

**FVB/N**

**BALB/c**

**A**

MEVs NPM - + + - + +

Myco - - + - - +

BALB/c

FVB/N

**Figure S5**. **Genes induced during the first exposure to *M. ulcerans* vesicles with purified mycolactone in BALB/c macrophages.** Macrophages from BALB/c and FVB/N mice were seeded into plates and incubated for six hours +/- vesicles from a mycolactone deficient strain (MEVs NPM, MOI: 20,000) +/- purified mycolactone (Myco, 6 ng/mL). mRNA was collected and gene expression was measured by RNAseq. (A) Differential gene expression summary comparing cells stimulated with MEVs NPM + Myco vs. cells stimulated only MEVs NPM only, for each mouse strain. Genes induced have log2FC >= 2, Q value <= 0.05. (B) Heatmap of 9 genes induced in BALB/c macrophages only highlighted in Fig. 4A. Data are based on three independent replicates.
