# Supplementary material for: Type-I interferons promote innate immune tolerance in macrophages exposed to Mycobacterium ulcerans vesicles
Source: PLoS Pathog. 2023 Jul 10;19(7):e1011479. doi: 10.1371/journal.ppat.1011479 (PMC10358927; doi:10.1371/journal.ppat.1011479)
Supplement: S6 Fig — BALB/c and FVB/N mice were injected in the tail with living M. ulcerans organisms. Mice were sacrificed at the ulcerative stage and the skin from tails was collected. mRNA was collected and gene expression was measured by RNAseq. (A) Heatmap of the genes induced in infected BALB/c mice only as compared to control (log2FC > = 1.5, Q value < = 0.05). (B) Pathway enrichment of genes induced only in infected BALB/c mice. the Rich ratio is the ratio of the number of differentially expressed genes annotated in this pathway relative to all genes annotated in this pathway. A Q value is the corrected p value ranging from 0 to 1. Q values < 0.05 are considered significant. (DOCX) [file ppat.1011479.s006.docx]

**Figure S6**. **Genes induced during infection with *M. ulcerans* in BALB/c mice only.** BALB/c and FVB/N mice were injected in the tail with living *M. ulcerans* organisms. Mice were sacrificed at the ulcerative stage and the skin from tails was collected. mRNA was collected and gene expression was measured by RNAseq. (A) Heatmap of the genes induced in infected BALB/c mice only as compared to control (log2FC >= 1.5, Q value <= 0.05). (B) Pathway enrichment of genes induced only in infected BALB/c mice. the Rich ratio is the ratio of the number of differentially expressed genes annotated in this pathway relative to all genes annotated in this pathway. A Q value is the corrected p value ranging from 0 to 1. Q values < 0.05 are considered significant.
